# Coordinative structures as scale-free networks: Cascade and percolation dynamics in motor learning with empirical validation

**DOI:** 10.1371/journal.pcbi.1014523

**Published:** 2026-07-21

**Authors:** Chulwook Park

**Affiliations:** 1 Department of Physical Education, Seoul National University, Seoul, South Korea; 2 Systemic Risk and Resilience, International Institute for Applied Systems Analysis (IIASA), Laxenburg, Austria; 3 Complexity Science and Evolution, Okinawa Institute of Science and Technology (OIST), Okinawa, Japan; La Jolla Institute for Immunology, UNITED STATES OF AMERICA

## Abstract

Coordinative structures are the functional groupings of degrees of freedom that simplify motor control. Decades of research have documented them well, yet they remain mechanistically unexplained. How they emerge and why they are hierarchically organized are both unresolved questions. This study proposes scale-free network topology as the missing mechanism. Systematic simulations compared random networks, small-world networks, and scale-free networks. Scale-free organization reproduced the defining features of coordinative structures more completely than either alternative. Those features are a hub-periphery hierarchy, ordered hub-first recruitment, an abrupt onset of global coordination, and a balance between stability and flexibility. Four formal correspondences connect these features to established coordination phenomena. A coupled learning model reproduced the characteristic curve of skill acquisition, and scale-free networks reached the coordination threshold fastest. The model was then validated against published data from five independent studies spanning motor learning, bimanual coordination, brain networks, and joint coordination. It yields eleven testable predictions with explicit quantitative thresholds, together with five criteria that would disconfirm it. Network topology therefore offers a principled account of how coordinative structures form through structural constraints and experience. That account is empirically grounded and open to falsification.

## Author summary

Learning a new motor skill requires coordinating hundreds of muscles into smooth and purposeful movement. The body organizes muscles into functional groups called coordinative structures, yet how these groups form has remained unclear. This study represents the connections between degrees of freedom as a scale-free network in which a few hub elements coordinate many peripheral ones. That arrangement accounts for the properties coordinative structures display. The simulations show that hub elements propagate coordination signals far faster than peripheral elements. They also show that coordination emerges through sharp threshold transitions and that practice strengthens hub connections preferentially. These results were compared with published findings from five independent studies of motor learning, bimanual coordination, brain network dynamics, and joint coordination. All simulation code and validation data are openly archived for verification. The model generates eleven testable predictions with explicit quantitative thresholds, and five disconfirmation criteria keep the account falsifiable. Each criterion targets a distinct mechanistic link, so any failure identifies what requires revision instead of discrediting the model as a whole. The account could therefore guide how practice and rehabilitation are designed, and it gives a shared basis for describing how human movement is organized.

## 1. Introduction

### 1.1. Bernstein’s problem and coordinative structures

The human motor system presents a fundamental computational challenge known as Bernstein’s degrees of freedom (DOF) problem [1]. The body comprises approximately 600 skeletal muscles that must coordinate to produce purposeful movement, resulting in roughly 100 mechanical DOF and a 200-dimensional state space when position and velocity are both considered [2,3]. Bernstein’s insight was that the nervous system does not control individual DOF independently but instead organizes them into functional groupings termed “coordinative structures,” now commonly called motor synergies or muscle synergies [1,4]. These task-specific assemblies reduce the number of independent variables requiring explicit central nervous system (CNS) control, with empirical studies consistently showing that 4–8 muscle synergies account for the majority of variance in activation patterns across diverse motor tasks [5,6].

Coordinative structures exhibit a dual nature, they provide standardization for consistent, reproducible movements while preserving flexibility for adaptation to varying conditions. Schmidt’s schema theory [7] and subsequent work on generalized motor programs (GMPs) formalized this observation, demonstrating that well-learned movements maintain relative timing invariance across speed variations while permitting parameter scaling of duration, amplitude, and force [7,8]. At the neural level, Kawato and colleagues [9] proposed that the CNS acquires internal models of movement dynamics through cerebellar learning circuits. In their account, the spinocerebellum develops a forward dynamics model that predicts movement outcomes, while the cerebrocerebellum acquires an inverse dynamics model that computes required motor commands directly from desired trajectories. This hierarchical architecture provides a neurophysiological basis for understanding how coordinative structures emerge through learning and enable progressive automation of complex motor skills.

Motor learning research has identified characteristic progressions in DOF organization during skill acquisition. Vereijken and colleagues [10] documented a freezing-freeing-exploiting sequence: novices initially constrain DOF to simplify control, subsequently release DOF as coordination improves, and ultimately exploit DOF interactions to optimize performance. This progression suggests that coordinative structure formation follows systematic developmental trajectories mirroring the hierarchical acquisition of internal models described in computational motor control theory [3,9–11]. Despite substantial progress in describing these phenomena, a fundamental gap persists. No mechanistic account adequately explains how coordinative structures emerge, why they exhibit hierarchical organization, or what determines their characteristic stability-flexibility balance. Dynamic systems approaches [12,13] have emphasized self-organization but often lack quantitative predictive power, while computational approaches [9] have identified neural architectures without fully specifying the network-level principles governing coordination emergence. This theoretical gap motivates the present work.

### 1.2. Network science foundations

*Scale-free topology in biological systems.* Network science has revealed that biological systems across multiple scales share a common organizational principle, namely scale-free topology characterized by power-law degree distributions $P(k)\propto k^{\left(-\gamma \right)}$, where γ represents node connectivity and typically ranges between 2 and 3 [14,15]. Unlike random networks with homogeneous connectivity, scale-free networks exhibit pronounced heterogeneity in which a small number of highly connected hub nodes coexist with numerous peripheral nodes of low connectivity. This architecture pervades biological organization. Metabolic networks across 43 organisms from all three domains of life display conserved scale-free topology [16]. Protein-protein interaction networks follow a centrality-lethality rule whereby hub proteins are disproportionately essential for organism viability [17], with a temporal distinction between hubs that coordinate simultaneous interactions and those that connect modules sequentially [18]. The human brain exhibits analogous organization, with hub regions in posterior medial, parietal, and lateral prefrontal cortices serving as integration centers for distributed neural processing [19,20]. These hubs form a densely interconnected rich-club core [21] that consumes disproportionate metabolic resources and shows heightened vulnerability to pathological processes [22].

*Cascade dynamics and spreading phenomena.* Scale-free topology fundamentally transforms how perturbations propagate through networks. In epidemic spreading models, the critical threshold for widespread propagation depends on network structure, and for scale-free networks with γ<3, this threshold approaches zero: $\beta /\mu >\left\langle k\right\rangle /\left\langle k^{\text{2}}\right\rangle \to \text{0}$, where β is the transmission rate, μ is the recovery rate, and ⟨k⟩ and $\left\langle k^{\text{2}}\right\rangle$ denote the first and second moments of the degree distribution [23,24]. Hub nodes function as “super-spreaders,” with hub-initiated cascades propagating faster, often by an order of magnitude or more, than those initiated at peripheral nodes, creating pronounced cascade asymmetry [25,26]. Scale-free networks also exhibit a characteristic “robust-yet-fragile” property: random node removal has minimal impact on global connectivity because most removed nodes are low-degree peripherals, whereas targeted hub removal rapidly fragments the network [27–29]. This dual character emerges directly from the heterogeneous degree distribution and has been observed in systems ranging from the Internet to cellular and neural networks.

*Percolation theory and phase transitions.* Percolation theory provides a rigorous frame for phase transitions in network connectivity [30,31]. The percolation threshold $p_{c}$ defines the critical fraction of occupied bonds at which a giant component spanning the system first emerges. Formally, the giant component is the connected subgraph whose size scales with the number of nodes *N*. Below the threshold, every component contains only O(logN) nodes and occupies a vanishing fraction of the network as N→∞; above it, a single component contains O(N) nodes and occupies a finite fraction of the system. The emergence of this extensive component at $p_{c}$ is the structural event that defines the percolation transition. For scale-free networks with γ<3, Cohen and colleagues [32] demonstrated that $p_{c}\to \text{0}$, meaning that sparse hub connectivity alone can generate system-wide integration. Although the threshold itself vanishes, the phase transition remains sharp: a small change in connectivity around the critical point produces a disproportionately large, sharp change in global connectivity [32,33]. This combination of low threshold and sharp transition predicts that gradual, incremental changes in connectivity can produce sudden qualitative shifts in system-wide coordination, consistent with the plateau-followed-by-breakthrough pattern commonly observed in motor skill acquisition.

**Fig 1:**
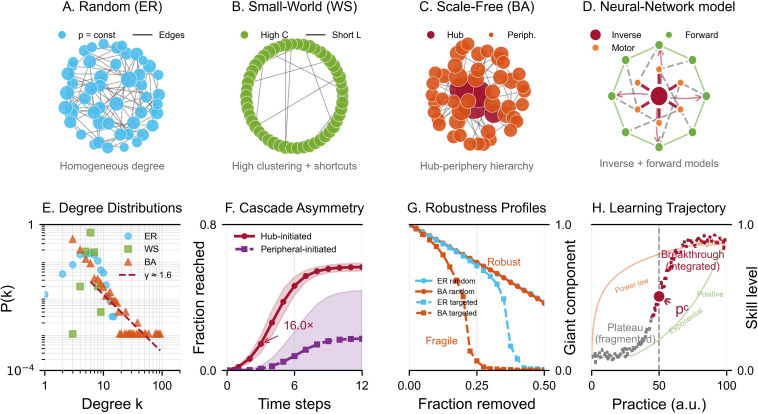
Network topologies, hierarchical neural architecture, and cascade dynamics. **(A)** Random network (Erdős–Rényi), homogeneous degree distribution from constant connection probability, node size proportional to degree. **(B)** Small-world network (Watts–Strogatz), high clustering and short path length, drawn as a ring with rewired shortcuts. **(C)** Scale-free network (Barabási–Albert), power-law degree distribution and hub-periphery hierarchy, the five highest-degree hubs in dark red. **(D)** Hierarchical neural-network model after Kawato, an inverse dynamics model as the central hub linked to motor output nodes (inner ring) and forward dynamics models (outer ring) providing feedback. **(E)** Degree distributions on log-log axes, Poisson for ER, bounded for WS, and power-law for BA with fitted exponent γ ≈2.9. **(F)** Cascade propagation asymmetry, hub-initiated cascades spreading faster than peripheral-initiated ones, shaded regions marking ±1 SD across 20 trials. **(G)** Robustness under random failure versus targeted attack, scale-free networks tolerating random failures but fragmenting rapidly under targeted hub removal. **(H)** Learning trajectory, the sigmoid curve (solid line) marking the percolation threshold at which fragmented skill components integrate into coherent coordinative structures, with exponential and power-law curves for reference.

*Comparative network topology.* To establish why scale-free topology, rather than random or small-world alternatives, best reproduces the properties of coordinative structures, we systematically compare three canonical network types in the Materials and Methods (Section 2.1): Erdős–Rényi (ER) random networks [34,35], Watts–Strogatz (WS) small-world networks [36], and Barabási–Albert (BA) scale-free networks [14]. ER networks exhibit structural homogeneity with no hub-periphery organization, producing uniform cascade propagation incompatible with the hierarchical coordination observed in motor systems. WS networks, constructed by rewiring a fraction of the edges of a regular ring lattice [37], achieve high clustering with short path lengths but retain approximately Poisson degree distributions, lacking the pronounced hub structure required for cascade asymmetry. Of the three, only BA networks, through their growth-with-preferential-attachment mechanism, generate power-law degree distributions, hub-peripheral cascade asymmetry [25,26], and robust-yet-fragile properties [27,29] that, among the topologies compared, most closely match the observed properties of coordinative structures. These three topologies, their structural measures, and their dynamical signatures are visualized in Fig 1 (with detailed comparison in Table A in S1 Appendix).

### 1.3. Research purpose and core thesis

Despite decades of research on coordinative structures and motor synergies, five phenomena still lack an adequate mechanistic explanation. The first is how initially independent degrees of freedom (DOF) become functionally coupled during learning. The second is why coordinative structures exhibit hierarchical organization, in which dominant leading elements [38] coordinate subordinate ones rather than forming flat, egalitarian coupling. The third is the principled basis for the stability-flexibility balance, whereby relative timing remains invariant while scaling parameters adapt. The fourth is how perturbations propagate through coordinative structures and why disruptions to some elements produce more severe consequences than others, a question for which cascade dynamics perspectives [23,39] have not been systematically integrated with motor control theory. The fifth is why motor learning follows characteristic nonlinear trajectories, with plateaus followed by sudden breakthroughs [40] whose timing and magnitude remain unexplained.

The present work addresses these gaps by proposing that coordinative structures can be formally modeled as scale-free networks, with cascade dynamics and percolation threshold theory providing mechanistic explanations for their emergence, hierarchy, and learning trajectories. This study pursues five specific aims. The first (i) is to demonstrate, through systematic comparison, that scale-free topology reproduces the properties of coordinative structures more completely than random and small-world alternatives. The second (ii) is to establish formal correspondences between scale-free network properties and coordinative structure phenomena, thereby generating quantitative predictions. The third (iii) is to integrate cascade propagation dynamics as a mechanistic basis for the spread of perturbations through coordinative structures. The fourth (iv) is to incorporate percolation threshold theory in order to explain the nonlinear phase transitions observed in motor learning. The fifth (v) is to generate testable predictions that distinguish the scale-free model from alternative theoretical accounts.

The model rests on four formal correspondences, each mapping a well-characterized network property to an observed coordination phenomenon, building upon and extending computational approaches to motor learning [9]. Preferential attachment maps to freezing-freeing dynamics, explaining how newly activated DOF couple preferentially to dominant synergies [3,10,14]. Power-law degree distributions map to neuromuscular hierarchy, linking hub-periphery structure to the leading joint hypothesis [38] and the finding that 4–8 muscle synergies capture most activation variance [5,6]. The robust-yet-fragile property maps to GMP stability, explaining tolerance for peripheral parameter variations alongside vulnerability to disruption of core timing relationships [7,8,27,29,41]. Cascade asymmetry maps to motor learning trajectories, connecting faster hub-initiated propagation [25,26] to the characteristic progression from rapid initial coordination gains to gradual refinement [9,40,42,43]. Percolation threshold theory unifies these correspondences by identifying DOF reduction, hierarchy, stability-flexibility balance, and learning trajectories as interrelated consequences of threshold-crossing dynamics in scale-free networks [30–32]. For γ<3, $p_{c}\to \text{0}$ predicts that sparse hub connectivity suffices to trigger sudden integration of previously fragmented DOF clusters, accounting for the plateau-followed-by-breakthrough pattern in skill acquisition [40]. The formal specifications of all four correspondences, the percolation model, and the coupled learning dynamics are developed in Materials and Methods.

## 2. Materials and methods

### 2.1. Network model specifications

We implement three canonical network models to enable precise mechanistic comparison: Erdős–Rényi (ER) random, Watts–Strogatz (WS) small-world, and Barabási–Albert (BA) scale-free networks. Each encodes a distinct hypothesis about coordinative structure emergence. All implementations use custom code, validated source code is available in the GitHub repository.

The ER random network uses the G(N,p) model, connecting each pair of N nodes with independent probability p, producing Poisson-distributed degrees with mean ⟨k⟩=p(N−1) and serving as the null hypothesis baseline. The WS small-world network constructs a ring lattice where node *i* connects to (i+s) mod *N* for s∈{1,…,K/2}, then rewires each edge with probability β, preserving high clustering while introducing shortcut connections (see S1 Appendix for implementation details). The BA scale-free network generates power-law degree distributions through growth with degree-proportional connection probability:


$$\begin{array}{c} \prod \left(i\right)=k_{i}/\sum_{j}k_{j} \end{array}$$ (1)

where $k_{i}$ is the degree of node *i*. Starting from a complete graph of $m_{\text{0}}=m$ nodes, each new node connects to m existing nodes selected with probability proportional to their current degrees (Eq 1), yielding $P(k)\propto k^{\left(-\gamma \right)}$ with γ→3 and producing the hub-periphery hierarchy central to our model [44]. Targets are sampled without replacement (see S1 Appendix for algorithmic details).

All three networks use N=100 nodes and comparable mean degree ⟨k⟩≈6, achieved with ER p=0.0606, WS K=6 and β=0.1, and BA m=3. Despite identical connectivity budgets, the topologies differ fundamentally in degree heterogeneity, as quantified in Table B in S1 Appendix. Ensemble statistics are computed over 100 independent realizations with fixed random seed = 42; sensitivity analyses across parameter ranges are reported in Table C in S1 Appendix.

Two structural measures derived from these ensembles serve as the primary tests of hub-periphery hierarchy, and their rationale is stated here because they recur throughout the Results. The first is degree heterogeneity, $\kappa =\left\langle k^{\text{2}}\right\rangle /\left\langle k\right\rangle$, the ratio of the second to the first moment of the degree distribution. Because all three topologies are matched on mean degree (⟨k⟩≈6), mean connectivity cannot distinguish them, so κ isolates the spread of the distribution and separates a hub-organized network from a homogeneous one. Rather than an arbitrary score on which a larger value is simply taken to be preferable, κ is the specific structural quantity that governs the network’s dynamics, since it sets the percolation threshold (Eq 6, equivalently $p_{c}=\text{1}/(\kappa -\text{1})$) and diverges with network size for scale-free degree distributions (2<γ≤3) [32]. A higher κ in the BA model is therefore meaningful because it indicates that coordinating connectivity is concentrated in a few hub degrees of freedom rather than spread evenly, the structural substrate for the leading-element hierarchy that defines a coordinative structure [3,38]. The second measure is the Gini coefficient of eigenvector centrality, which quantifies inequality of dynamical influence across nodes and ranges from zero for uniform influence to values approaching one when influence is concentrated in a few nodes. Whereas κ captures heterogeneity in raw connectivity, the eigenvector-centrality Gini captures heterogeneity in propagated influence, so the two measures test the same hierarchy property from complementary structural and dynamical perspectives. A high Gini indicates a small set of dominant leading degrees of freedom coordinating many subordinate ones, the network signature of hierarchical coordinative organization.

### 2.2. Cascade dynamics model

We model activation propagation through network structure by adapting network-agent propagation models from systemic risk analysis [45,46] to the motor coordination domain. The dynamics are reframed as DOF recruitment cascades with hub DOFs exhibiting higher stability that ensures hierarchical recruitment order, preserving the mathematical structure of the original models while aligning with Bernstein’s freezing-freeing concept. Detailed derivations and parameter sensitivity analyses are provided in S2 Appendix.

Each node *i*, representing a single DOF, is characterized by four state variables, the activation potential $\varphi_{i}(t)\in \left\{\text{0},\text{1}\right\}$, activation state $A_{i}(t)\in \left\{\text{0},\text{1}\right\}$, the eigenvector centrality $C_{i}\in [\text{0},\text{1}]$, and the coordination strength $s_{i}(t)\geq \text{0}$. Eigenvector centrality assigns each node an influence score proportional to the sum of its neighbors’ scores, so it reflects not only how many connections a degree of freedom has but how well connected those neighbors are. Formally, $C_{i}$ is the *i*-th entry of the leading eigenvector *x* of the adjacency matrix *A*, obtained from the eigenvalue equation $A_{x}=\lambda_{x}$ and normalized to its maximum value, so that the most central degree of freedom equals one and the remaining values lie in [0,1] [15] (S2 Appendix). For a connected network with a nonnegative adjacency matrix, the Perron–Frobenius theorem guarantees that this leading eigenvector is unique and strictly positive, so $C_{i}$ is well defined and positive for every degree of freedom [15]. This makes eigenvector centrality a natural predictor of cascade behavior, because activation at a high-$C_{i}$ hub reaches well-connected neighbors and propagates rapidly through the network, whereas activation at a low-$C_{i}$ peripheral node remains localized. The hub-peripheral asymmetry $\Delta_{hp}$ defined below is the dynamical expression of this centrality difference. The binary potential model aligns with the all-or-none principle of motor unit recruitment and preserves correspondence with percolation theory’s discrete state transitions. The cascade proceeds through three mechanisms, spontaneous activation with probability $p_{n}$ per timestep, neighbor-mediated propagation with probability $p_{l}$ per link from active DOFs, and stability-dependent state transition. The stability (protection probability) $\pi_{i}$ is defined as


$$\begin{array}{c} \pi_{i}=p_{max}/\left[\text{1}+c_{p}/\left(f_{\pi_{i}}\times s_{i}\right)\right] \end{array}$$ (2)

and the activation probability given potential is


$$\begin{array}{c} P\left(A_{i}=\text{1}|\varphi_{i}>\text{0}\right)=\text{1}-\pi_{i} \end{array}$$ (3)

where $p_{max}$ is the maximum stability, $c_{p}$ is a reference constant, and $s_{i}$ is coordination strength. The stability fraction $f_{\pi_{i}}$ incorporates eigenvector centrality:


$$\begin{array}{c} f_{\pi_{i}}=f_{\text{0}}+f_{\text{1}}\times C_{i} \end{array}$$ (4)

where $f_{\text{0}}$ is baseline stability and $f_{\text{1}}$ weights the centrality contribution. To avoid confusion between symbols that both appear in the stability function, $C_{i}$ is the node-specific eigenvector centrality and $f_{\pi_{i}}$ is the centrality-weighted stability fraction, whereas $c_{p}$ (lowercase *c*, fixed subscript *p*) is a single global reference constant (Table A in S2 Appendix) that sets the coordination strength at which protection reaches half of its maximum. Hub DOFs with high $C_{i}$ values exhibit higher stability, resisting activation until coordination strength accumulates and ensuring controlled sequential activation consistent with the freezing-freeing progression. Upon activation, coordination strength resets ($s_{i}\leftarrow \text{0}$) and the DOF propagates activation to its neighbors; between activations, strength accumulates as $s_{i}(t+\text{1})=\text{1}+(\text{1}-f_{m}-f_{\pi_{i}})\times s_{i}(t)$.

Three measures quantify how cascade dynamics differ across topologies: early-stage propagation rate $R_{early}=\left|A(t=\tau )\right|/|A(\text{0})|$ within the first τ=5 timesteps, cascade reach $R_{\infty }=\underset{t\to \infty }{\text{lim}}\left|A(t)/N\right|$ indicating coordination completeness, and hub-peripheral asymmetry $\Delta_{hp}=R_{early}(hub)/R_{early}(peripheral)$ quantifying hierarchical organization. Simulations use fixed stability parameters ($f_{\text{0}}=f_{\text{1}}=\text{0}.\text{7}$) and propagation parameters ($p_{n}=p_{l}=\text{0}.\text{1}$), with 100 independent realizations and 100 cascade trials per realization. Table A in S2 Appendix summarizes all parameters; implementation code is available in the GitHub repository.

### 2.3. Mapping network topology to coordinative structures

This section establishes four formal correspondences between scale-free network properties and coordinative structure phenomena (Table 1). Each constitutes a structural isomorphism generating testable predictions. Percolation threshold theory [28,32] provides the unifying bridge, determining when hubs emerge, hierarchy crystallizes, and cascade dynamics shift from localized to system-wide propagation. Detailed derivations and simulation protocols are provided in S3 Appendix.

**Table 1:** Four correspondences mapping scale-free topology to coordinative structures.

Network property	Coordination phenomenon	Formal mechanism	Testable prediction
Preferential attachment	Freezing-freeing dynamics	Fitness-extended BA kernel with percolation threshold	Hub formation lowers coordination threshold; phase-transition onset
Power-law degree distribution	Neuromuscular hierarchy	$P(k)\propto k^{\left(-\gamma \right)}$, hub count $\propto N^{\left(\text{1}/(\gamma -\text{1})\right)}$	Leading joints scale as power law; first 3–5 synergies capture >80% variance
Robustness-fragility trade-off	GMP stability	$f_{r}$(targeted)>> $f_{r}$(random); asymmetry ratio $A_{r}$	Hub perturbation: disproportionate breakdown; Aᵣ higher in skilled performers
Cascade asymmetry	Motor learning trajectories	$\Delta_{hp}=\frac{R_{early}(hub)}{R_{early}(peripheral)}$	Rapid early organization via hubs; gradual peripheral refinement

Note. BA = Barabási–Albert; GMP = generalized motor program; P(k) = degree distribution; γ = power-law exponent; *N* = number of nodes; $f_{r}$ = fragmentation ratio after node removal; $A_{r}$ = robustness asymmetry ratio (targeted vs. random removal); $\Delta_{hp}$ = hub-peripheral cascade asymmetry; $R_{early}$ = early-stage propagation rate. Each correspondence is formally specified in Section 2.3 (Eqs 5–8).

*Correspondence 1: Preferential attachment and freezing-freeing dynamics.* In Bernstein’s framework [3], learners initially freeze redundant DOFs, then progressively free them as coordination develops. We propose that this progression follows preferential attachment: newly freed DOFs couple preferentially to established coordinative hubs. To incorporate task-specific demands, we extend the BA attachment kernel with a fitness parameter [47]:


$$\begin{array}{c} \pi_{i}=\left(k_{i}^{\alpha }\times \eta_{i}^{\beta }\right)/\sum_{j}\left(k_{j}^{\alpha }\times \eta_{j}^{\beta }\right) \end{array}$$ (5)

where $\eta_{i}$ is biomechanical fitness representing task relevance, α controls rich-get-richer strength, and β weights task demands relative to connectivity history (reducing to the standard BA model when α=1, β=0). The percolation threshold $p_{c}$, the fraction of active bonds at which a giant connected component first appears [28,32], is


$$\begin{array}{c} p_{c}=\left\langle k\right\rangle /\left(\left\langle k^{\text{2}}\right\rangle -\left\langle k\right\rangle \right) \end{array}$$ (6)

For scale-free networks with 2<γ≤3, $\left\langle k^{\text{2}}\right\rangle$ diverges with network size, driving $p_{c}\to \text{0}$. Hub formation via preferential attachment thus lowers the threshold for coordination emergence, producing the plateau-then-breakthrough trajectory observed in skill acquisition [48,49] (S3 Appendix, Part B).

*Correspondence 2: Power-law distribution and neuromuscular hierarchy.* The degree distribution $P(k)\propto k^{\left(-\gamma \right)}$ creates hub-periphery hierarchy. For BA networks with γ≈3, an N=100 network contains approximately 3–5 hub nodes while most nodes maintain near-average connectivity [44]. This maps onto the leading joint hypothesis [38], the finding that 3–5 principal components explain more than 80% of multi-muscle electromyography (EMG) variance [5,50], and Kawato’s hierarchical control architecture [9] in which hub DOFs correspond to slow invariant parameters and peripheral DOFs to fast context-dependent adjustments.

*Correspondence 3: Robustness-fragility trade-off and GMP stability.* Scale-free networks exhibit strong tolerance to random node removal but acute vulnerability to targeted hub removal [27]. The fragmentation ratio is


$$\begin{array}{c} f_{r}(q)=\text{1}-S(q)/S(\text{0}) \end{array}$$ (7)

where S(q) is the largest connected component after removing fraction *q* of nodes, and the robustness asymmetry ratio $A_{r}(q)=f_{r}(targeted,\;q)/f_{r}(random,\;q)$. This maps onto GMP stability [7,8]: invariant features (relative timing, relative force) correspond to hub DOF coordination, while variant parameters correspond to peripheral adjustments. Hub perturbation should cause disproportionate breakdown, and $A_{r}$ should be greater for skilled than novice performers (S3 Appendix, Part C).

*Correspondence 4: Cascade asymmetry and motor learning trajectories.* Hub-initiated cascades propagate faster than peripheral-initiated cascades (Cascade Dynamics Model). When a cascade initiates from a hub with degree $k_{hub}$, the activated set grows approximately as


$$\begin{array}{c} |A(t)|\approx |A(\text{0})|\times {\left(\text{1}+p_{l}\times k_{hub}\right)}^{t} \end{array}$$ (8)

This maps onto motor learning, rapid early organization occurs as hub DOFs recruit peripheral DOFs at accelerating rates, explaining the initial phase of the power law of practice [43,51]. Gradual peripheral refinement follows via localized cascades; and skill transfer correlates with hub DOF overlap through the minimum intervention principle [52] (S3 Appendix, Part F).

The four correspondences form an interlocking model unified by the percolation threshold. Preferential attachment (Eq 5) generates the power-law distribution creating hub-periphery hierarchy, which produces the robustness-fragility trade-off (Eq 7) and cascade asymmetry (Eq 8). The percolation threshold (Eq 6) determines when each property transitions from latent to expressed. Simulation validation is presented in S3 Appendix.

### 2.4. Percolation model and coupled learning dynamics

We define the coordinative structure network $G_{CS}=(V,E,W)$ where $V=\left\{V_{\text{1}},\;\ldots ,V_{n}\right\}$ represents ${DOF}_{S}$, E⊆V×V represents functional couplings, and $W:E\to R^{+}$ represents coupling strengths that evolve through learning (see S4 Appendix for detailed derivations).

The percolation threshold $p_{c}$ (Eq 6) determines when coordination emerges; the dynamics model characterizes how the transition unfolds. The bond occupation probability $p(t)=\left|E(t)\right|/|E_{max}|$ increases with practice. The order parameter is the giant component fraction:


$$\begin{array}{c} p_{\infty }(p)=\left|C_{\text{1}}(p)\right|/N \end{array}$$ (9)

Here $C_{\text{1}}(p)$ denotes the largest connected component, the giant component, of the network at bond-occupation level *p*, and *N* is the total number of nodes. The order parameter $p_{\infty }$ is therefore the fraction of nodes belonging to the giant component, so that $C_{\text{1}}(p)$ is the structural quantity whose emergence marks the percolation transition. Below pc the network fragments, with $p_{\infty }\approx \text{0}$, and above $p_{c}$ a giant component emerges, with $p_{\infty }>\text{0}$, marking the coordination breakthrough. Transition sharpness is quantified by the susceptibility


$$\begin{array}{c} {\chi (p)=dp}_{\infty }/dp \end{array}$$ (10)

Scale-free networks produce sharper transitions (higher $\chi_{max}$, lower $p_{peak}$) than ER or WS networks, predicting more abrupt coordination breakthroughs [30]. Simulations sweep p∈[0,1] in increments of 0.01 across 100 realizations per topology (S4 Appendix).

Motor skill acquisition is formalized as a six-step coupled algorithm that integrates Sections 2.1 to 2.3 with Hebbian weight updates. At each practice iteration, a new DOF is added via fitness-extended preferential attachment (Eq 5), bond occupation p(t) and the giant component $p_{\infty }(t)$ are computed (Eq 9), activation spreads through $G_{CS}$ via the cascade model (Eqs 2–4), and edge weights update via


$$\begin{array}{c} W_{ij}(t+\text{1})=W_{ij}(t)+\eta \times A_{i}(t)\times A_{j}(t)-\delta \times W_{ij}(t) \end{array}$$ (11)

where η is the learning rate, $A_{i}(t)$ and $A_{j}(t)$ are activation states (Cascade Dynamics Model), and δ is a decay term preventing unbounded growth [53,54]. Edges between co-activated hub nodes strengthen fastest, reinforcing scale-free topology through a positive feedback loop: hubs attract connections (Eq 5), resist perturbation (Eq 7), propagate cascades efficiently (Eq 8), and accumulate the strongest weights (Eq 11). Convergence occurs when ${\left\|W(t+\text{1})-W(t)\right\|}_{F}<\varepsilon$ and degree heterogeneity $\kappa =\left\langle k^{\text{2}}\right\rangle /\left\langle k\right\rangle$ stabilizes. Table A in S4 Appendix summarizes all percolation and coupled learning parameters; sensitivity analyses are reported in S4 Appendix; implementation code is available in the GitHub repository.

### 2.5. Empirical validation data sources

To evaluate whether the model’s predictions correspond to observed motor behavior, we compared simulation outputs against published empirical data from five independent sources spanning different motor tasks, measurement modalities, and timescales. The empirical data were used as validation benchmarks for three sets of model predictions, presented in Figs 4–6.

For network topology validation (Fig 4A), we reconstructed coordination network architectures from three published studies representing each canonical topology. Vereijken and colleagues [10] documented frozen bilateral DOF coupling in novice ski-simulator performance, yielding a uniformly connected structure consistent with ER topology. Bassett and colleagues [55] reported modular brain functional connectivity during motor sequence learning, exhibiting small-world properties consistent with WS topology. Scholz and Schöner [56] demonstrated uncontrolled manifold (UCM) variance decomposition in sit-to-stand transitions, revealing a center-of-mass hub dominating the joint hierarchy consistent with BA topology. Reconstruction parameters, coupling thresholds, and topology-matching criteria are documented in S4 Appendix, Part D.

For learning trajectory and phase transition validation (Fig 5), two empirical datasets provided independent tests. Liu, Mayer-Kress, and Newell [40] tracked performance and variability across extended practice of a roller ball coordination task, enabling comparison with the model’s predicted three-phase sigmoid trajectory (plateau–bifurcation–stabilization). Kelso, Scholz, and Schöner [57] documented critical fluctuations at the coordination phase transition in bimanual oscillation, enabling comparison with the model’s predicted susceptibility peak at $p_{c}$. Data extraction procedures for both panels are detailed in S4 Appendix, Part C.

For predicted outcome validation (Fig 6), the eleven quantitative predictions generated by the model (Predicted Empirical Outcomes) were mapped against existing empirical findings from the motor control literature. The validation sources, selected based on publication in peer-reviewed journals, direct relevance to specific predictions, and methodological compatibility with proposed experimental protocols, are documented in S5 Appendix.

### 2.6. Target properties of coordinative structures and their network signatures

Sections 2.1–2.4 specified the network models, cascade dynamics, formal correspondences, and coupled learning dynamics, and the preceding section identified the empirical data against which the model’s predictions are compared. Before turning to the results, we consolidate the coordinative-structure properties that the model is required to reproduce, so that every result in the following sections can be read as a direct test of a specific property rather than as an isolated statistic. We consider five properties established in the motor-control literature: hub-periphery hierarchy [3,38], hierarchical recruitment [10], the stability-flexibility balance of generalized motor programs [7,8], sharp threshold-like emergence of global coordination [40], and experience-dependent differentiation of core couplings [53]. Each is operationalized by a distinct, independently measurable network signature with an associated statistic (Table 2).

**Table 2:** Target properties of coordinative structures and their network signatures.

Property	Network measure	Rationale	Statistic
Hub-periphery hierarchy	κ, EC Gini, hub fraction, γ^	Isolates degree-distribution spread at matched mean degree	Ensemble mean ± SD, BA vs. ER and WS t-tests; KS goodness-of-fit for γ^
Hierarchical recruitment	$\Delta_{hp}$	Quantifies hub-first propagation order	Ensemble mean ± SD; fraction of realizations with $\Delta_{hp}$ > 2
Stability-flexibility balance	$A_{r}$	Contrasts targeted-hub vs. random tolerance	Asymmetry ratio at *q* = 0.28 (targeted vs. random removal)
Sharp coordination emergence	$p_{c}$, $p_{peak}$, $X_{max}$, $\Delta_{p}$	Captures abruptness of giant-component onset	Threshold at $p_{\infty }$ = 0.5; susceptibility peak and height; transition width ($p_{\infty }$ from 0.1 to 0.9)
Experience-dependent differentiation	$H_{W}$, $t_{\text{0}.\text{5}}$	Tracks preferential consolidation of core couplings	Hub-hub vs. peripheral weight ratio after practice; iterations to $p_{\infty }$ = 0.5

Note. EC = eigenvector centrality; $\kappa =\left\langle k^{\text{2}}\right\rangle /\left\langle k\right\rangle$ (degree heterogeneity); hub fraction = proportion of nodes with Ce>μ+σ;γ^ = MLE power-law exponent; $\Delta_{hp}$ = hub-peripheral cascade asymmetry; $A_{r}$ = robustness asymmetry ratio; $p_{c}$, $p_{peak}$, $X_{max}$, $\Delta_{p}$= percolation transition measures; $H_{W}$ = hub-hub vs. peripheral weight ratio; $t_{\text{0}.\text{5}}$ = iterations to $p_{\infty }$ = 0.5. Ensemble statistics are computed over 100 realizations (*N* = 100, ⟨k⟩ ≈ 6, seed = 42); learning measures derive from the 1000-iteration trajectory. Measure definitions appear in Sections 2.1 to 2.4.

The rationale column states in brief why each measure is diagnostic of its property. The full justification is developed in Sections 2.1 to 2.4, which explain in particular why degree heterogeneity κ, rather than mean connectivity, distinguishes the topologies. A property is treated as reproduced only when its signature is present in the scale-free (BA) topology and is statistically distinguishable from the random (ER) and small-world (WS) baselines. This criterion makes explicit whether scale-free topology is specifically required by comparison with the two alternatives.

### 2.7. Computational implementation and statistical analysis

This study is entirely computational. No human or animal participants were involved, and no ethics approval was required. All simulations were implemented in Python 3.9 using NetworkX 2.8, NumPy 1.23, and Matplotlib 3.6. Reproducibility was ensured through fixed random seeds (seed = 42 for all primary analyses) and explicit specification of all simulation parameters in S1–S4 Appendices. Ensemble statistics were computed over 100 independent network realizations per condition and are reported as means ± standard deviations. Statistical comparisons between network topologies used two-sided independent-samples *t*-tests at a significance level of α=0.05, together with Cohen’s *d* as a measure of effect size and Bonferroni correction for multiple comparisons across topology pairs. The *t*-statistic, corrected *p*-value, and Cohen’s *d* for each comparison are reported in Table B in S5 Appendix. Source code and simulation scripts are available at https://github.com/pcw8531/Coordinative-structures-scale-free-networks and are archived on Zenodo with the persistent DOI https://doi.org/10.5281/zenodo.20694466. The repository structure and reproduction steps are described in S6 Appendix.

## 3. Results

The following sections test, in turn, the five target properties defined in Section 2.6 (Table 2). Each subsection reports the network measure for that property across the three topologies, with the measure’s definition cross-referenced to Materials and Methods.

### 3.1. Network topology comparison

Section 3.1 tests the hub-periphery hierarchy property, using the structural measures defined in Section 2.1. The three network ensembles produced structurally distinct topologies despite matched mean connectivity. The key differentiating measure is degree heterogeneity $\kappa =\left\langle k^{\text{2}}\right\rangle /\left\langle k\right\rangle$: BA networks yield κ=9.67±0.59, approximately 42% higher than ER (6.84) and 59% higher than WS (6.09). Power-law fitting confirmed γ^=2.78±0.15 with Kolmogorov–Smirnov (KS) goodness-of-fit test p=0.33, consistent with theoretical predictions for finite BA networks (Table B in S1 Appendix; complete measures in Table D in S1 Appendix) (Table 3).

**Table 3:** Consolidated structural, dynamical, and learning measures across network topologies.

Measure	ER (Random)	WS (Small-World)	BA (Scale-Free)
$\kappa =\left\langle k^{\text{2}}\right\rangle /\left\langle k\right\rangle$	6.84 ± 0.38	6.09 ± 0.02	9.67 ± 0.59
γ^(MLE)	—	—	2.78 ± 0.15
$\Delta_{hp}$	2.25	1.46	3.60 ± 0.62
$p_{peak}$	0.22	0.27	0.16
$A_{r}$	1.14 ± 0.09	1.02 ± 0.05	2.92 ± 0.31
$H_{W}$	0.5	0.4	5.5
$t_{\text{0}.\text{5}}$ (steps)	84	262	56

*Note.* N=100, ⟨k⟩≈6, seed = 42, 100 realizations. κ = degree heterogeneity; γ^ = Maximum Likelihood Estimation (MLE) power-law exponent; $\Delta_{hp}$ = hub-peripheral cascade asymmetry ratio at τ = 5; $p_{peak}$ = peak susceptibility bond occupation; $A_{r}$ = robustness asymmetry ratio at *q* = 0.28; $H_{W}$ = weight hierarchy index (${\overset{―}{W}}_{hub-hub}/{\overset{―}{W}}_{periph-periph}$); $t_{\text{0}.\text{5}}$ = practice iterations to $p_{\infty }$ = 0.5. Complete ensemble data in Table D in S1 Appendix, Table B in S2 Appendix, Table A in S3 Appendix, and Table B in S4 Appendix.

**Fig 2:**
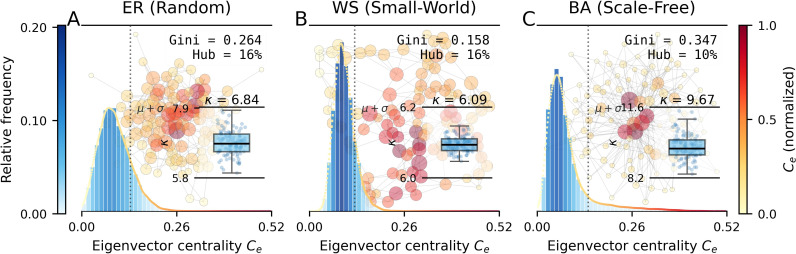
Integrated structural validation of network topologies. Each panel overlays an exemplar network (*N* = 100, seed = 42; node size proportional to degree, node color encoding eigenvector centrality $C_{e}$ via the YlOrRd colormap, right colorbar) with the $C_{e}$ distribution pooled across 100 realizations, shown as relative-frequency bars (left colorbar) with an overlaid dotted kernel-density curve colored by $C_{e}$. Inset annotations report the Gini coefficient and hub fraction; inset box-and-scatter plots show the κ ensemble distribution. **(A)** ER random network: moderate centrality dispersion with Gini = 0.264 and κ = 6.84. **(B)** WS small-world network: most homogeneous distribution with Gini = 0.158 and κ = 6.09. **(C)** BA scale-free network: highest centrality inequality with visible hub-periphery hierarchy, Gini = 0.347 and κ = 9.67.

The eigenvector centrality Gini coefficient revealed a clear gradient: WS (0.16) <ER (0.26) <BA (0.35). Hub identification using the criterion $C_{e}>\mu +\sigma$ yields a smaller hub fraction in BA networks (10%) than in ER or WS (both 16%), reflecting extreme concentration of influence in fewer nodes consistent with coordinative hierarchy [1,3]. Fig 2 integrates these structural properties.

### 3.2. Cascade dynamics

**Fig 3:**
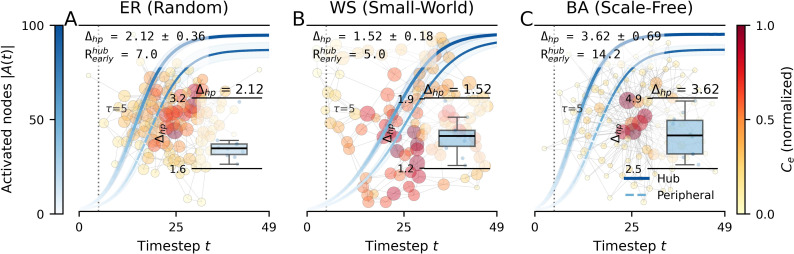
Cascade dynamics across network topologies. Panels A–C show cascade trajectories |A(t)| over 50 timesteps for hub-initiated (solid lines) and peripheral-initiated (dashed lines) cascades, with ±1 SD ribbons across 100 trials. Background network visualizations display exemplar topology with node color encoding $C_{e}$ (YlOrRd colormap) and node size proportional to degree. Vertical dotted line marks τ=5. Annotations report $\Delta_{hp}$ and ${R_{early}}^{hub}$ values. Inset boxplots show the ensemble $\Delta_{hp}$ distribution across 100 realizations. **(A)** ER random network: $\Delta_{hp}=\text{2}.\text{1}\text{2}\pm \text{0}.\text{3}\text{6}$ with moderate trajectory separation. **(B)** WS small-world network: $\Delta_{hp}=\text{1}.\text{5}\text{2}\pm \text{0}.\text{1}\text{8}$ with minimal trajectory separation. **(C)** BA scale-free network: $\Delta_{hp}=\text{3}.\text{6}\text{2}\pm \text{0}.\text{6}\text{9}$ with marked hub-peripheral trajectory divergence and $\Delta_{hp}>\text{2}.\text{0}$ across all realizations. Figure annotations reflect per-figure realization parameters; ensemble statistics cited in text are based on 100-realization means (Table B in S2 Appendix).

Section 3.2 tests hierarchical recruitment, using the cascade measures defined in Section 2.2. Cascade dynamics (Eqs 2–4) were evaluated using topology-isolated trials with single-seed initiation and no spontaneous activation [45]. Hub-initiated cascades in BA networks achieved $R_{early}=\text{1}\text{4}.\text{1}\text{5}\pm \text{1}.\text{9}\text{7}$ at τ=5, approximately 1.9 times higher than ER (7.58) and 2.8 times higher than WS (4.98), reflecting the hub connectivity advantage predicted by Eq 8. The hub-peripheral asymmetry ratio $\Delta_{hp}$ provided the signature test for hierarchical cascade organization: BA networks yield $\Delta_{hp}=\text{3}.\text{6}\text{0}\pm \text{0}.\text{6}\text{2}$, substantially exceeding ER (2.25) and WS (1.46). Critically, BA is the only one of the three topologies where $\Delta_{hp}$ consistently exceeds 2.0 across all 100 realizations (range: 2.04–5.57), identifying hierarchical cascade organization as a reliable signature that distinguishes the scale-free model (Fig 3). In motor control terms [3,4], this asymmetry predicts that hub DOFs rapidly organize coordination across the network while peripheral DOF recruitment proceeds incrementally. Full ensemble statistics are reported in Table B in S2 Appendix.

### 3.3. Percolation dynamics and robustness-fragility

**Fig 4:**
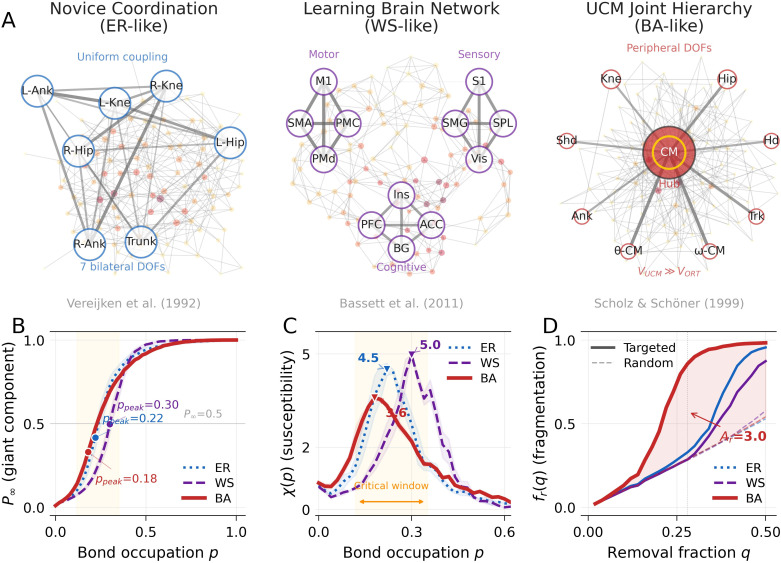
Percolation dynamics and robustness-fragility across network topologies. **(A)** Empirical coordinative structures illustrating topology-task correspondences. Left: novice ski-simulator inter-joint coupling network (n=7 bilateral lower-limb and trunk DOFs) showing near-uniform connectivity characteristic of ER-like “frozen” coordination (Vereijken et al., 1992). Center: motor sequence learning brain network (n=12 cortical regions) showing modular organization with cross-module shortcuts characteristic of WS-like architecture (Bassett et al., 2011). Right: UCM sit-to-stand joint hierarchy (n=9 variables) showing center-of-mass (CM) as dominant hub stabilizing task-level performance ($V_{UCM}\gg V_{ORT}$), characteristic of BA-like scale-free organization (Scholz & Schöner, 1999). **(B)** Giant component fraction $p_{\infty }(p)$ overlaid for all topologies (ER: dotted blue; WS: dashed purple; BA: solid red; ± 1 SD ribbons) with $p_{peak}$ markers and critical transition zone (amber). **(C)** Susceptibility $\chi (p)=dp_{\infty }/dp$ with peak values annotated; orange bracket indicates critical window. **(D)** Robustness-fragility profiles under targeted (solid) versus random (dashed) node removal; shaded region highlights BA divergence with $A_{r}$ annotation. Figure annotations reflect 30-realization visual parameters; quantitative values cited in text are based on 100-realization ensemble statistics (Tables A and B in S3 Appendix).

Section 3.3 tests two target properties, sharp coordination emergence and the stability-flexibility balance, using the percolation and robustness measures defined in Sections 2.3 and 2.4. Progressive bond occupation sweeps (p∈[0,1], step 0.01, 100 realizations) revealed topology-dependent percolation transitions (Fig 4). BA networks crossed the $p_{\infty }=\text{0}.\text{5}$ threshold at $p_{c}=\text{0}.\text{2}\text{0}\text{8}\pm \text{0}.\text{0}\text{2}\text{3}$, compared with 0.221 for ER and 0.244 for WS, consistent with the theoretical prediction that elevated κ lowers $p_{c}$ (Eq 6). The susceptibility χ(p) peak occurred at $p_{peak}=\text{0}.\text{1}\text{6}$ for BA, earlier than ER (0.22) or WS (0.27), indicating that the coordination transition initiates at lower connectivity density. BA networks also exhibited the steepest rise, with $p_{\infty }$ increasing from 0.10 to 0.90 over a narrower range (Δp≈0.22 versus 0.28 for ER and 0.32 for WS), predicting more abrupt coordination breakthroughs consistent with the plateau-then-sudden-improvement pattern in motor learning [7,8].

Node removal simulations (Eq 7; S3 Appendix) produced the most pronounced topological distinction. Under random removal at *q* = 0.28, all topologies showed comparable fragmentation ($f_{r}\approx$ 0.28–0.29). Targeted hub removal, however, produced $f_{r}(targeted)$ = 0.84 ± 0.09 for BA versus 0.33 for ER and 0.29 for WS, yielding a robustness asymmetry ratio $A_{r}=\text{2}.\text{9}\text{2}\pm$ 0.31 — 2.6 times larger than ER and 2.9 times larger than WS. Among the three topologies, this robust-yet-fragile signature appeared only in the scale-free model, and did so across all 100 realizations (range: 1.65–3.32): peripheral DOF perturbations leave the giant component intact, preserving GMP invariant timing [7], whereas hub disruption fragments the coordinative network.

Empirical coordination networks ground these percolation predictions in published movement data (Fig 4A). Novice ski-simulator inter-joint coupling [10] produces near-uniform connectivity (coupling range 0.60–0.85) with no differentiated hub structure, characteristic of ER-like “frozen” coordination. Motor sequence learning brain networks [55] exhibit modular organization linked by sparse cross-module shortcuts, characteristic of WS architecture. UCM analysis of sit-to-stand joint coordination [56] reveals center-of-mass as a dominant hub ($V_{UCM}\gg V_{ORT}$), characteristic of BA-like scale-free topology.

The cumulative evidence chain thus links structural hierarchy (κ≈10) to cascade asymmetry ($\Delta_{hp}=\text{3}.\text{6}\text{0}$), which supports both earlier coordination emergence ($p_{peak}=\text{0}.\text{1}\text{6}$) and the robust-yet-fragile property ($A_{r}=\text{2}.\text{9}\text{2}$) mapping directly onto GMP stability.

### 3.4. Coupled learning dynamics

Section 3.4 tests experience-dependent differentiation, using the coupled learning dynamics defined in Section 2.4. The coupled algorithm (Eq 11) was executed for T=1000 practice iterations per topology, with Hebbian learning rate η=0.01 and decay δ=0.001 (Table A in S4 Appendix). In BA networks, hub-hub edge weights grew to $\overset{―}{W}\approx \text{2}\text{5}$ while peripheral-peripheral edges remained near $\overset{―}{W}\approx \text{2}$, producing a weight hierarchy $H_{W}=\text{5}.\text{5}$ — compared with 0.5 for ER and 0.4 for WS. Values below 1.0 for ER and WS indicate that, among the three topologies compared, coordinative differentiation emerged only in the scale-free model.

**Fig 5:**
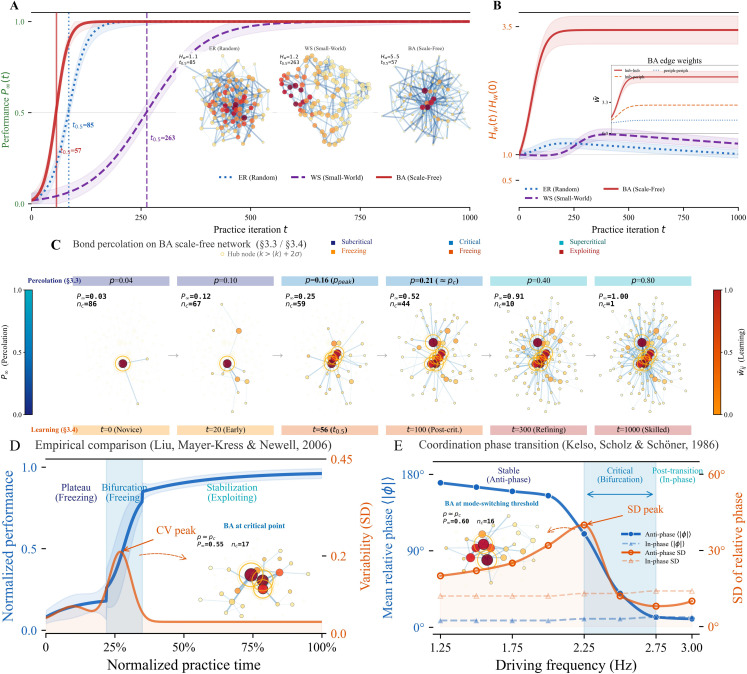
Coupled learning dynamics across network topologies. **(A)** Performance trajectories $p_{\infty }(t)$ with ±1 SD ribbons; vertical lines mark $t_{\text{0}.\text{5}}$ (BA = 56, ER = 84, WS = 262); insets show converged topologies colored by $C_{e}$. **(B)** Weight hierarchy $H_{W}(t)/H_{W}(\text{0})$; BA maintains hierarchy while ER and WS decay. Inset: edge weight evolution by class. **(C)** Bond percolation filmstrip on BA network mapping percolation onto Hebbian learning. Six snapshots (*p* = 0.04–0.80) show giant component growth from fragmentation through hub-mediated coalescence to full connectivity; nodes colored by $C_{e}$, hubs marked with gold rings. Top/bottom bars: percolation phases and corresponding learning stages; colorbars: normalized $p_{\infty }$ and ${\overset{―}{W}}_{ij}$. **(D)** Empirical comparison to Liu, Mayer-Kress, and Newell (2006): normalized performance (blue) and variability (orange) against practice time. Phase labels mark plateau/freezing, bifurcation/freeing, and stabilization/exploiting stages; shading highlights the bifurcation zone. Variability peaks at bifurcation, consistent with Prediction 3. Inset: BA network at $p\approx p^{c}$. **(E)** Coordination phase transition from Kelso, Scholz, and Schöner (1986): mean relative phase ⟨|φ|⟩ (blue) and *SD* (orange) versus driving frequency. Anti-phase coordination destabilizes with critical fluctuation enhancement (*SD* peak) before abrupt transition to in-phase. Inset: BA network at mode-switching threshold. Figure annotations reflect per-figure realization parameters; ensemble statistics cited in the text are based on 100-realization means.

Performance $p(t)=p_{\infty }(t)$ exhibited the three-phase sigmoid pattern predicted by the correspondence model (Fig 5A). BA networks crossed the $p_{\infty }=\text{0}.\text{5}$ threshold at $t_{\text{0}.\text{5}}=\text{5}\text{6}$, reaching this milestone 33% faster than ER ($t_{\text{0}.\text{5}}=\text{8}\text{4}$) and 79% faster than WS ($t_{\text{0}.\text{5}}=\text{2}\text{6}\text{2}$). The normalized weight hierarchy $H_{W}(t)/H_{W}(\text{0})$ (Fig 5B) reveals learning-driven amplification: BA networks maintained hierarchy throughout 1000 iterations while ER and WS showed substantial decay ($H_{W}/H_{W}(\text{0})$ declining to 0.3–0.5). The underlying mechanism is visible in the edge weight evolution: hub-hub edges grew rapidly via degree-weighted Hebbian reinforcement, hub-peripheral edges stabilized at intermediate values ($\overset{―}{W}\approx \text{8}$), and peripheral-peripheral edges grew slowly. This three-class weight separation generates the hierarchical coordinative structure predicted by the correspondence model [1,3] and provides a mechanistic explanation for why scale-free organization, rather than the random or small-world alternatives, supports coordinative differentiation [4,5].

Two independent empirical datasets validate these coupled dynamics predictions. Liu, Mayer-Kress, and Newell [40] tracked performance and variability across extended practice in a roller ball task (Fig 5D): normalized performance exhibited the predicted three-phase sigmoid, and the coefficient of variation peaked sharply at the bifurcation point, confirming the critical fluctuation prediction on the learning timescale. Kelso, Scholz, and Schöner [57] documented complementary evidence in real-time coordination dynamics (Fig 5E): anti-phase bimanual coordination destabilized with critical fluctuation enhancement (*SD* peak) before abrupt transition to in-phase, demonstrating the same variability-peak-at-criticality signature across a fundamentally different timescale. Detailed data extraction procedures are provided in S4 Appendix.

### 3.5. Predicted empirical outcomes

Section 3.5 translates the preceding results into testable predictions. The preceding sections established a cumulative computational chain that reproduces the five target properties (Section 2.6, Table 2): structural hierarchy (κ≈10) generates cascade asymmetry ($\Delta_{hp}=\text{3}.\text{6}\text{0}$), which supports percolation-mediated coordination emergence ($p_{peak}=\text{0}.\text{1}\text{6}$) and coupled learning dynamics producing hierarchical weight consolidation ($H_{W}=\text{5}.\text{5}$, $t_{\text{0}.\text{5}}=\text{5}\text{6}$). This section translates these findings into eleven testable predictions with five disconfirmation criteria. The six most distinctive predictions with direct computational support are summarized in Table 4; the remaining five predictions (Predictions 2, 5, 8, 9, 11) with their full benchmarks are provided in Table A in S5 Appendix. Detailed empirical grounding and recommended experimental protocols for all eleven predictions are documented in S5 Appendix.

**Table 4:** Core predicted empirical outcomes with quantitative reference values and disconfirmation criteria.

#	Prediction	Model basis	Quantitative reference	Disconfirmation criterion
1	Hub perturbations propagate faster than peripheral	$\Delta_{hp}$ = 3.60 (Eqs 2–4, 8)	$R_{early}\;$ratio > 2.0 in 50–100 ms	$\Delta_{hp}$ ≈ 1.0: symmetric cascade
3	Critical fluctuations precede breakthroughs	$\chi_{max}$ at $p_{peak}$ = 0.16 (Eqs 9–10)	CV increase 10–20 trials pre-breakthrough	No variability peak before transition
4	Three-phase sigmoid learning	$p_{\infty }(t)$ sigmoid, $t_{\text{0}.\text{5}}$ = 56 (Eq 11)	Rapid phase ≈ 5–10% of practice	Strictly monotonic learning curve
6	Hub-hub coupling consolidates first	$H_{W}$ = 5.5; $\overset{―}{W}(hh)$ ≈ 25 (Eq 11)	Hub-hub coherence leads by ≥1 phase	Uniform coupling growth ($H_{W}$ ≈ 1.0)
7	Skilled networks show power-law p(k)	γ^ = 2.78, κ = 9.67 (Eq 1)	2<γ<3; KS *p* > 0.05	Poisson/exponential preferred (LRT *p* < 0.05)
10	Transfer correlates with hub overlap	T(A,B) overlap (Eq 5; S3 Appendix Part c)	Positive correlation (*r* > 0.4)	Transfer independent of T(A,B)

*Note.* Six core predictions shown; Predictions 2, 5, 8, 9, and 11 with full reference values are provided in Table A in S5 Appendix. Each prediction derives from simulation results mapped through the four correspondences (Table 1). Hub defined as degree> ⟨k⟩+2σ; peripheral defined as degree <⟨k⟩, LRT = Likelihood Ratio Test.

**Fig 6:**
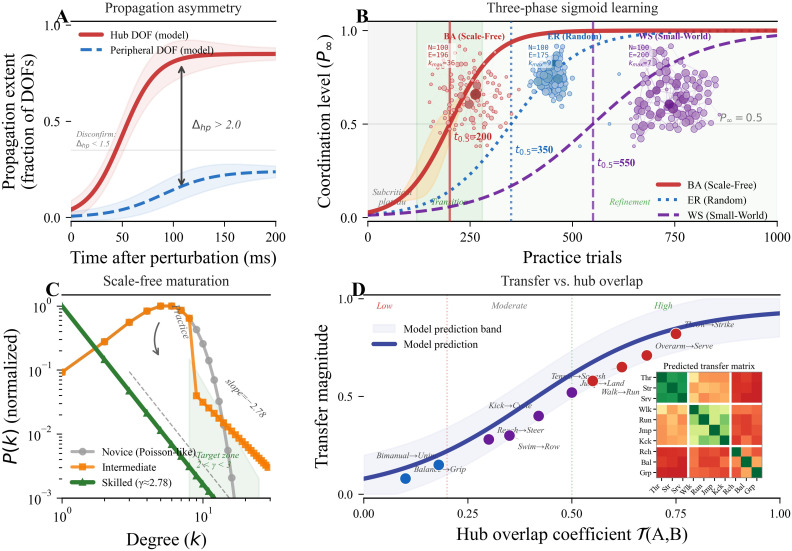
Predicted empirical outcomes. Panel A: Predicted perturbation propagation asymmetry — hub DOF (solid red) versus peripheral DOF (dashed blue) cascade trajectories with ±1 SD prediction bands; $\Delta_{hp}>\text{2}.\text{0}$ benchmark annotated at t=100 ms; gray line indicates disconfirmation threshold ($\Delta_{hp}<\text{1}.\text{5}$). Prediction 1. Panel B: Three-phase sigmoid learning trajectory with topology comparison: BA (solid red), ER (dotted blue), WS (dashed purple); phase shading marks subcritical plateau (gray), rapid transition (amber), and refinement (green); vertical dotted lines mark t₀.₅; inset bar chart compares t₀.₅ across topologies; bottom row shows representative network visualizations. Prediction 4. Panel C: Degree distribution maturation from novice (gray, Poisson-like) through intermediate (orange) to skilled (green, γ≈2.78) on log-log axes; 2<γ<3 target zone shaded. Prediction 7. Panel D: Transfer magnitude versus hub overlap coefficient T(A,B) with sigmoid prediction curve and ±1 SD band; task pair scatter points color-coded by overlap level; inset 6 × 6 hub overlaps heatmap. Prediction 10.

The cascade dynamics analysis yields Prediction 1: hub DOF perturbations should propagate to 5–30 times more coupled elements within 50–100 ms than equivalent peripheral perturbations, reflecting the $\Delta_{hp}=\text{3}.\text{6}\text{0}\pm \text{0}.\text{6}\text{2}$ asymmetry where hub-initiated cascades achieved $R_{early}$ = 14.15 versus 3.99 for peripheral initiation (Fig 6A). A complementary prediction (Prediction 2; Table A in S5 Appendix) specifies that hub perturbation recovery should require 3–5 times longer than peripheral recovery. Trial-to-trial variability should increase 10–20 trials before coordination breakthroughs and decrease sharply afterward (Prediction 3), mirroring peak susceptibility $\chi_{max}$ at $p_{peak}$ — a pattern receiving preliminary support from both Liu et al.’s roller ball data (Fig 5D) and Kelso et al.’s bimanual oscillation data (Fig 5E).

The coupled learning dynamics generate Prediction 4: learning curves should exhibit a three-phase sigmoid comprising a subcritical plateau (approximately 5–15% of total practice), a rapid transition (approximately 5–10%), and a refinement phase (75–90%), with BA networks reaching $p_{\infty }=\text{0}.\text{5}$ fastest (Fig 6B). Hub-hub coupling should consolidate during the transition phase while peripheral coupling develops gradually during refinement, with$\;H_{W}$ reaching 5.5 (Prediction 6). Additional predictions regarding κ-learning rate correlation (Prediction 5) and network maturation with practice (Prediction 8) are documented in Table A in S5 Appendix.

The structural validation and learning dynamics jointly predict that skilled coordination networks should exhibit power-law degree distributions with 2<γ<3 (Prediction 7; Fig 6C), and that hub DOFs identified via eigenvector centrality should overlap more than 60% with synergy leaders from principal component analysis (PCA), non-negative matrix factorization (NMF), or uncontrolled manifold (UCM) analysis (Prediction 9; Table A in S5 Appendix). The transfer model predicts that skill transfer should correlate with the hub overlap coefficient $T(A,B)=\left|H_{A}\cap H_{B}\right|/\left|H_{A}\cup H_{B}\right|$ (Prediction 10; Fig 6D), with high overlap (T>0.5) predicting positive transfer and asymmetric transfer favoring hub-strengthening training (Prediction 11; Table A in S5 Appendix).

Five disconfirmation criteria ensure falsifiability, each targeting a necessary condition of a distinct mechanistic link. (1) Symmetric cascade propagation ($\Delta_{hp}<\text{1}.\text{5}$) would disconfirm hierarchical propagation. (2) Non-scale-free coordination networks (KS p<0.05) would disconfirm the topological basis. (3) Strictly monotonic learning trajectories would disconfirm the percolation account. (4) Hub-independent transfer patterns would disconfirm the cascade-based transfer model. (5) Uniform weight consolidation ($H_{W}\approx \text{1}.\text{0}$) would disconfirm Hebbian differentiation. Observing any one of these outcomes identifies the specific mechanistic link that needs revision, allowing targeted refinement rather than rejection of the whole model.

## 4. Discussion

### 4.1. Summary of findings

This study examined whether scale-free network topology provides a mechanistic account of coordinative structures in motor control. Through systematic computational comparison of Erdős–Rényi random [34], Watts–Strogatz small-world [36], and Barabási–Albert scale-free [14] networks, the results indicate that scale-free topology most consistently reproduces the full set of properties characteristic of coordinative structures [1]. These properties comprise hub-periphery organization with confirmed power-law scaling (γ^=2.78±0.15) [58], cascade asymmetry ($\Delta_{hp}=\text{3}.\text{6}\text{0}$) [23,39], a robustness-fragility trade-off ($A_{r}=\text{2}.\text{9}\text{2}$) [32], and a lowered percolation threshold ($p_{peak}=\text{0}.\text{1}\text{6}$) [30]. Coupled learning dynamics produced hierarchical weight consolidation ($H_{W}=\text{5}.\text{5}$) and three-phase sigmoid learning trajectories that reached coordination thresholds faster than either alternative topology. Four formal correspondences (Table 1), namely preferential attachment to freezing-freeing dynamics [3], power-law distributions to neuromuscular hierarchy [5], robustness-fragility to GMP stability [7], and cascade asymmetry to learning trajectories [40], were computationally verified and unified by the percolation threshold. The analysis yielded eleven testable predictions with quantitative thresholds and five disconfirmation criteria, positioning the model for empirical refinement.

### 4.2. Theoretical implications

The cascade dynamics provide a structural account of hierarchical coordination. In BA networks, hub-initiated cascades propagated at $R_{early}=\text{1}\text{4}.\text{1}\text{5}$ compared with 3.99 for peripheral initiation ($\Delta_{hp}=\text{3}.\text{6}\text{0}\pm \text{0}.\text{6}\text{2}$), a magnitude not approached by either alternative topology. This aligns with Dounskaia’s leading joint hypothesis [38], in which proximal joints generate interaction torques that drive distal joint motion [59]. Hub DOFs serve an analogous role, in which high connectivity ensures rapid cascade propagation through the mechanism of Eqs 2–4, consistent with the empirical finding that the shoulder generates 60–80% of total mechanical power during horizontal arm swings [60]. The cascade asymmetry also offers a topological basis for the minimum intervention principle [52]. In optimal feedback control, task-relevant variables receive active correction while task-irrelevant variability is tolerated. Valero-Cuevas and colleagues [61] confirmed this pattern empirically. In the scale-free network model, equivalent selective protection emerges from centrality-dependent stability (Eq 4), where hub perturbations propagate system-wide while peripheral perturbations remain localized, without requiring explicit cost optimization.

The percolation analysis addresses the nonlinear dynamics of skill acquisition. BA networks reached peak susceptibility at $p_{peak}=\text{0}.\text{1}\text{6}$ with the most compressed transition profile ($\Delta_{p}\approx \text{0}.\text{2}\text{2}$), mapping onto the plateau-then-breakthrough pattern documented by Vereijken and colleagues [10] in ski-simulator learning and by Gray [62] in baseball batting, where coupling between adjacent swing phases increased abruptly with training. Below threshold, added couplings benefit only isolated clusters. At threshold crossing, the giant component emerges and coordination becomes globally integrated, producing the qualitative change that Liu, Mayer-Kress, and Newell [40] documented as a bifurcation in motor learning dynamics.

The Hebbian weight evolution (Eq 11), grounded in Hebb’s co-activation principle [53] and formalized experimentally as spike-timing-dependent plasticity [54], produced three-class weight separation in BA networks, with hub-hub edges consolidating to $\overset{―}{W}\approx \text{2}\text{5}$, hub-peripheral edges stabilizing at $\overset{―}{W}\approx \text{8}$, and peripheral-peripheral edges remaining near $\overset{―}{W}\approx \text{2}$. Alternative topologies did not produce $H_{W}$ values exceeding 1.0, indicating that hierarchical weight consolidation depends critically on the degree heterogeneity that scale-free topology provides. This converges with neuroimaging evidence. Van den Heuvel and Sporns [19] identified rich-club architecture in which hub-hub connections are preferentially strengthened, and Tomasi and Volkow [22] confirmed that connectivity hubs exhibit the strongest long-range connections. At the motor control level, the observed weight hierarchy maps onto Kawato’s hierarchical neural network model [9], with hub DOFs corresponding to invariant planning parameters and peripheral DOFs to context-dependent execution adjustments. Extending the model to multi-level coupled networks [63] remains an important theoretical direction. These coupled processes are formalized as a unified performance model.


$$\begin{array}{c} p(t)=p_{\infty }\left(W(t)\right)=\left|C_{\text{1}}\left(W(t)\right)\right|/N \end{array}$$ (12)

where $W(t)=\{W_{ij}(t)|W_{ij}(t)>W_{thresh}\}$, $W_{ij}(t)=f[A_{i}(t),\;A_{j}(t);\;\eta ,\delta ]$, and $A_{i}(t)=g[k_{i},\;C_{i};\;\tau ,\;f_{\text{0}},\;f_{\text{1}}]$. Here, $C_{\text{1}}\left(W(t)\right)$ is the largest connected component of the weight-thresholded network at practice iteration *t*, *f* implements the Hebbian update (Eq 11) driven by co-activation states, and *g* captures the cascade dynamics (Eqs 2–4) as determined by degree $k_{i}$ and eigenvector centrality $C_{i}$. Eq 12 formalizes the central theoretical contribution, namely that coordination performance is not imposed externally but emerges from the recursive interaction of topology-dependent cascade dynamics and experience-dependent weight evolution. The three-phase sigmoid (Fig 5A), weight hierarchy, and percolation transition (Fig 4B) are all consequences of this recursive process, with scale-free topology providing the degree heterogeneity (κ≈10) and cascade asymmetry ($\Delta_{hp}=\text{3}.\text{6}\text{0}$) that drive the compressed transition profile observed empirically [40].

### 4.3. Relationship to existing approaches

The dynamical systems approach [12,13] showed that coordinative patterns emerge through self-organization and undergo phase transitions when a control parameter crosses a critical value, as captured mathematically in the Haken–Kelso–Bunz (HKB) model [64]. The percolation analysis shares this foundation. The bond occupation probability *p* functions as a control parameter, and giant component emergence at $p_{c}$ constitutes a connectivity phase transition. The critical fluctuation enhancement predicted by dynamical systems theory [12] corresponds to the susceptibility maximum $\chi_{max}$ in the simulations. Fig 5E illustrates this directly, with the $\chi_{max}$ peak in Kelso, Scholz, and Schöner’s [57] bimanual data mapping onto the percolation susceptibility peak, and the same variability-peak-at-criticality signature appears on the learning timescale in Liu and colleagues’ data (Fig 5D), suggesting that this critical signature reflects network topology rather than the timescale of the control parameter. Where the dynamical systems approach characterizes transitions through collective variables and potential landscapes, the network model specifies the structural substrate that determines which transition profiles are possible and why certain DOFs lead the transition.

The uncontrolled manifold hypothesis [4] partitions variability into components that stabilize and components that destabilize task-relevant performance variables [56]. The network model offers a structural interpretation in which hub DOFs form the giant component backbone and are predicted to correspond to the controlled manifold basis, because perturbations that preserve hub connectivity leave performance (Eq 12) intact, producing the variability ($V_{UCM}>V_{ORT}$) pattern that Latash and colleagues observed [4]. Prediction 9 (Table A in S5 Appendix) formalizes this as a testable overlap between hub DOFs and synergy leaders. The muscle synergy literature provides the closest empirical parallel. D’Avella, Saltiel, and Bizzi [5] showed that four to five time-varying synergies capture the majority of variance in reaching, Ivanenko and colleagues [6] identified five basic patterns across walking conditions, and Ting and Macpherson [50] demonstrated that a limited synergy set suffices for postural control. In each case, certain muscles serve as functional leaders while others contribute more weakly across modules, a leader-follower organization that maps onto the hub-periphery distinction. The Hebbian weight evolution (Eq 11) provides a learning mechanism for how such modular structure consolidates with practice, and the conservation of synergy patterns across locomotor conditions [6] is consistent with a topology-dependent organizational principle.

Optimal feedback control [52] specifies what the motor system achieves through cost minimization, whereas the network model specifies how the underlying structure constrains and enables that achievement. The robust-yet-fragile property ($A_{r}=\text{2}.\text{9}\text{2}$) naturally generates selective hub protection consistent with the minimum intervention principle without requiring explicit optimization. A joint formulation in which optimal control operates on the weight-thresholded network (Eq 12) could unify these perspectives. A distinguishing feature of the present contribution is its emphasis on modular falsifiability. The dynamical systems approach generates qualitative predictions but rarely specifies quantitative disconfirmation criteria [12]. The uncontrolled manifold tradition provides powerful descriptive tools without yielding rejectable numerical criteria [4]. The muscle synergy approach identifies modules but leaves open why specific muscles become leaders [5]. Optimal control depends on adjustable cost functions [52]. The present model generates explicit predictions with five disconfirmation criteria, each targeting a distinct mechanistic link, so that if any criterion fails, the specific theoretical component requiring revision is identified rather than the entire account being invalidated.

### 4.4. Practical implications

The cascade asymmetry implies that training protocols should prioritize hub DOFs during early skill acquisition, since hub-initiated cascades propagate to substantially more coupled elements than peripheral-initiated cascades (see Fig A in S2 Appendix for a single-realization cascade on a representative scale-free network, including the degree-of-freedom activation-state matrix). This converges with Dounskaia’s leading joint hypothesis [38] and the empirical finding that learners initially freeze distal DOFs while establishing proximal control [10]. Kim, Hinrichs, and Dounskaia [60] provided further support showing proximal power dominance during multi-joint movement.

The three-phase sigmoid trajectory (Prediction 4; Fig 5D) suggests that instructors should anticipate and communicate the plateau phase explicitly. The subcritical plateau reflects necessary accumulation of sub-threshold hub-hub coupling before the percolation transition enables global coordination; misinterpreting this plateau as stagnation may lead to premature strategy changes. The timing of the rapid transition is governed by the degree heterogeneity κ of the task’s coordination network (Prediction 5; Table A in S5 Appendix). The robust-yet-fragile property provides a basis for distinguishing hub from peripheral pathology in rehabilitation. Peripheral DOF perturbation preserves the giant component, predicting that distal injuries should maintain the overall coordination pattern while reducing scaling precision [8]. Hub disruption produces catastrophic fragmentation, predicting that proximal joint pathology or neurological insult to high-centrality motor areas will necessitate coordination reconstruction rather than parametric adjustment. Prediction 3 formalizes the expectation that variability should increase before coordination breakthroughs during rehabilitation, as confirmed in both learning (Fig 5D) and real-time coordination (Fig 5E) paradigms.

**Fig 7:**
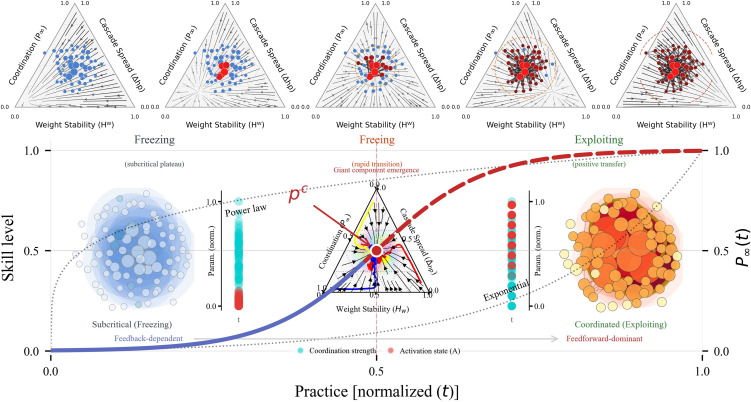
Integrative model of coupled learning dynamics on scale-free topology. The upper layer traces the coordination trajectory across practice with five ternary state-space plots, from the subcritical state through the percolation threshold to full coordination. Each simplex is spanned by three normalized coordination signatures, coordination (giant-component fraction), cascade spread, and weight stability, summing to one. Black streamlines show the flow field of the coupled dynamics. The scale-free network embedded in each simplex shows the structure at that stage, progressing from dispersed and dormant toward a connected, hub-dominated core. The bottom layer maps the same dynamics onto the freezing-freeing-exploiting model [10] against normalized practice. The three-phase sigmoid (Prediction 4) marks the feedback-to-feedforward transition at the percolation threshold, where the giant component emerges, bracketed by power-law and exponential reference curves. The central ternary inset situates this transition in the same state space, the trajectory moving from the subcritical to the high-coordination region at the breakthrough. Two network insets contrast its subcritical (left, dormant) and coordinated (right, activated) states, with node size proportional to degree and hubs strengthened on activation. The flanking strips plot the node-by-node averaged coordination strength (cyan) and activation state (red), each normalized to its maximum. The six-step coupled-dynamics flowchart and a single-realization cascade prototype appear in Fig A in S2 Appendix and Fig A in S4 Appendix, respectively.

The hub overlaps transfer coefficient $T(A,B)=\left|H_{A}\cap H_{B}\right|/\left|H_{A}\cup H_{B}\right|$ (Prediction 10) offers a principled basis for designing transfer-optimized training sequences. Tasks sharing hub DOFs (T(A,B)>0.5) should produce positive transfer, while low overlap (T(A,B)<0.2) would require independent coordination development. For talent development, the rate at which a young athlete’s coordination network matures toward scale-free organization, as indexed by increasing κ and $\Delta_{hp}$, could predict coordination capacity more reliably than early performance scores alone (Fig 7, see Fig A in S4 Appendix for the full computational pipeline integrating all six model stages).

### 4.5. Limitations and future directions

The model rests on simplifying assumptions that constrain its current predictions. Binary activation states ($A_{i}\in [\text{0},\text{1}]$), while justified by motor unit recruitment physiology and percolation theory correspondence (S2 Appendix), neglect the graded co-contraction patterns characteristic of real motor control [52]. Hogan [65] showed that impedance modulation through co-contraction plays a central role in limb stabilization. Extending the model to continuous states ($\varphi_{i}\in [\text{0},\text{1}]$) would enable modeling of smooth force-scaling properties that generalized motor programs exhibit [8]. The single-level network (N=100) does not capture the multi-scale organization from motor unit pools through synergies to kinematic chains [4,5]; multi-layer network models [63] would better represent this hierarchy. The uniform Hebbian learning rate η may underestimate consolidation differences arising from myelination [66] or attentional allocation [67], incorporating edge-specific rates $\eta_{ij}$ would improve weight hierarchy predictions. The empirical validation pathway presents methodological challenges. The predictions presuppose that coordination network topology can be reliably extracted from movement data, yet no standardized methodology exists. Candidate approaches include cross-correlation from multi-joint kinematics [10], partial directed coherence from EMG [68], and mutual information from motion capture [69]. Each imposes different coupling definitions and thresholds. The operational definition of a DOF varies across paradigms, and mapping simulation iterations to real practice time requires task-specific calibration. Until these methodological questions are resolved, quantitative benchmarks should be interpreted as ratio predictions (e.g., $\Delta_{hp}>\text{2}.\text{0}$, recovery time) rather than absolute values.

Several directions for future work emerge. The most immediate priority is experimental validation of the cascade asymmetry prediction (Prediction 1), requiring only cross-sectional perturbation data from a well-characterized multi-joint task with hub DOFs identified a priori from biomechanical analysis. A comprehensive validation program should pursue the eleven predictions in order of increasing methodological demand. On the theoretical side, replacing the static network with weighted, directed, and temporal representations [15,70] would enable modeling of asymmetric coupling and time-varying connectivity. Integrating the network approach with the HKB model [64] and UCM hypothesis [4] would unify the topological and dynamical vocabularies for coordination. A key step would be formalizing how the percolation threshold maps onto bifurcation points in coupled oscillator models, as suggested by Fig 5D–E. Incorporating individual differences in network topology from anatomical variation, prior experience, or developmental stage [11] would advance toward individualized models, addressing Wulf and Shea’s [71] observation that principles from simple skills do not always generalize to complex skill learning. Jeong and colleagues [16] showed that metabolic networks across 43 organisms exhibit scale-free organization, suggesting that the structural principles identified here may reflect conserved biological design. These extensions, combined with systematic empirical validation, would advance the framework toward a predictive tool for understanding and optimizing the network architecture of human coordination.

## Supporting information

S1 Appendix Network model specifications, parameters, and structural validation. Algorithmic specifications for ER, WS, and BA network construction; parameter justifications; power-law fitting methodology. Tables A–D.
(DOCX)

S2 Appendix Cascade dynamics model and parameters. Cascade propagation model derivations, stability function properties, and ensemble statistics. Tables A and B, Fig A.
(DOCX)

S3 Appendix Correspondence derivations, percolation, and robustness analysis. Formal correspondence derivations, percolation threshold theory, robustness-fragility protocol, and cascade-learning trajectory model. Tables A and B.
(DOCX)

S4 Appendix Coupled learning dynamics and empirical validation data sources. Hebbian weight evolution framework, percolation dynamics parameters, and data extraction procedures for Figs 4A and 5D–E. Tables A and B, Fig A.
(DOCX)

S5 Appendix Predicted empirical outcomes with benchmarks. Empirical grounding for eleven predictions with quantitative benchmarks, disconfirmation criteria, and suggested experimental protocols. Tables A and B.
(DOCX)

S6 Appendix Open-source code repository and reproduction guide. Repository structure, simulation scripts, empirical reference data, and reproduction instructions.
(DOCX)
