# Supplementary material for: Coordinative structures as scale-free networks: Cascade and percolation dynamics in motor learning with empirical validation
Source: PLoS Comput Biol. 2026 Jul 21;22(7):e1014523. doi: 10.1371/journal.pcbi.1014523 (PMC13423191; doi:10.1371/journal.pcbi.1014523)
Supplement: S1 Appendix — Algorithmic specifications for ER, WS, and BA network construction; parameter justifications; power-law fitting methodology. Tables A–D. (DOCX) [file pcbi.1014523.s001.docx]

## S1 Appendix. Network Model Specifications, Parameters, and Structural Validation

### Comparative properties of canonical network models

Table A summarizes the qualitative properties distinguishing the three canonical network topologies used throughout this study.

**Table A.** Comparative properties of canonical network models.

| Property | ER Random | WS Small-World | BA Scale-Free |
| --- | --- | --- | --- |
| Degree distribution | Poisson | ≈Poisson (bounded) | Power-law P(k) ∝ k⁻ᵞ |
| Hub structure | Absent | Absent | Pronounced hierarchy |
| Path length | ⟨l⟩ ∝ log N | ⟨l⟩ ∝ log N | ⟨l⟩ ∝ log N / log log N |
| Clustering | Vanishing (C → 0) | High (preserved) | Moderate |
| Cascade dynamics | Uniform propagation | Faster (shortcuts) | Asymmetric (hub-mediated) |
| Robustness pattern | Uniform degradation | Uniform degradation | Robust-yet-fragile |

### Network model parameters

All three networks use N = 100 nodes and comparable mean degree ⟨k⟩ ≈ 6. Ensemble statistics are computed over 100 independent realizations with fixed random seed = 42.

**Table B.** Network model parameters and topological properties (N = 100, seed = 42).

| Model | Parameters | ⟨k⟩ | σₖ | Clustering | Avg path |
| --- | --- | --- | --- | --- | --- |
| ER | p = 0.0606 | 6.0 | 2.4 | 0.06 | 2.5 |
| WS | K = 6, β = 0.1 | 6.0 | 1.1 | 0.47 | 4.2 |
| BA | m = 3 | 5.8 | 4.7 | 0.11 | 2.6 |

*Note.* σₖ = degree standard deviation. Ensemble averages over 100 realizations.

### Power-law fitting methodology

For discrete degree data with P(k) ∝ k⁻ᵞ, the MLE for γ is:

γ̂ = 1 + n [∑ᵢ ln(kᵢ / (k_min − 0.5))]⁻¹ (S1)

where n is the number of observations with k ≥ k_min [1]. Goodness-of-fit uses KS testing against synthetic power-law samples. For N = 100 BA networks: γ̂ = 2.9 ± 0.15 across 100 realizations, consistent with the theoretical prediction γ → 3 for the BA model.

**Table C.** Parameter sensitivity across network sizes (N = 50–500, ⟨k⟩ ≈ 6, 100 realizations). Reports ⟨k⟩, σₖ, κ, γ̂, and KS p-value for each topology at each network size.

| N | Topology | ⟨k⟩ | σₖ | κ | γ̂ (MLE) | KS p-value |
| --- | --- | --- | --- | --- | --- | --- |
| 50 | ER | 5.88 ± 0.46 | 2.25 ± 0.24 | 6.75 ± 0.52 | — | — |
| 50 | WS | 6.00 ± 0.00 | 0.82 ± 0.12 | 6.11 ± 0.03 | — | — |
| 50 | BA | 5.64 ± 0.00 | 3.58 ± 0.41 | 7.91 ± 0.68 | 3.12 ± 0.28 | 0.18 ± 0.15 |
| 100 | ER | 5.94 ± 0.33 | 2.31 ± 0.18 | 6.84 ± 0.38 | — | — |
| 100 | WS | 6.00 ± 0.00 | 0.75 ± 0.09 | 6.09 ± 0.02 | — | — |
| 100 | BA | 5.82 ± 0.00 | 4.72 ± 0.36 | 9.67 ± 0.59 | 2.78 ± 0.15 | 0.33 ± 0.18 |
| 200 | ER | 5.97 ± 0.24 | 2.38 ± 0.13 | 6.92 ± 0.28 | — | — |
| 200 | WS | 6.00 ± 0.00 | 0.71 ± 0.07 | 6.08 ± 0.01 | — | — |
| 200 | BA | 5.91 ± 0.00 | 6.41 ± 0.42 | 12.86 ± 0.95 | 2.85 ± 0.11 | 0.42 ± 0.16 |
| 500 | ER | 5.99 ± 0.15 | 2.42 ± 0.08 | 6.97 ± 0.17 | — | — |
| 500 | WS | 6.00 ± 0.00 | 0.68 ± 0.05 | 6.08 ± 0.01 | — | — |
| 500 | BA | 5.96 ± 0.00 | 9.87 ± 0.58 | 22.31 ± 1.82 | 2.91 ± 0.07 | 0.58 ± 0.14 |

*Note.* Ensemble means ± SD over 100 realizations. ER: p adjusted for ⟨k⟩ ≈ 6; WS: K = 6, β = 0.1; BA: m = 3. N = 100 values match Table D. κ = ⟨k²⟩/⟨k⟩ (degree heterogeneity). γ̂ and KS p-value reported only for BA (power-law fit). BA κ increases with N due to divergent second moment, confirming scale-free property.

### Structural validation measures

**Table D.** Structural validation measures (ensemble means ± SD; 100 realizations; N = 100, ⟨k⟩ ≈ 6, seed base = 42). κ = degree heterogeneity (⟨k²⟩/⟨k⟩; defined and motivated in Section 2.1 of the main text); γ̂ = MLE power-law exponent; Gini = Gini coefficient of eigenvector centrality; Hub fraction = proportion of nodes with Cₑ > μ + σ.

| Measure | ER (Random) | WS (Small-World) | BA (Scale-Free) |
| --- | --- | --- | --- |
| ⟨k⟩ | 5.94 ± 0.33 | 6.00 ± 0.00 | 5.82 ± 0.00 |
| σₖ | 2.31 ± 0.18 | 0.75 ± 0.09 | 4.72 ± 0.36 |
| k_max | 12.42 ± 1.42 | 8.18 ± 0.54 | 29.29 ± 4.60 |
| κ = ⟨k²⟩/⟨k⟩ | 6.84 ± 0.38 | 6.09 ± 0.02 | 9.67 ± 0.59 |
| γ̂ (MLE) | — | — | 2.78 ± 0.15 |
| KS p-value | — | — | 0.33 ± 0.18 |
| Gini (Cₑ) | 0.26 ± 0.02 | 0.16 ± 0.04 | 0.35 ± 0.01 |
| Hub fraction | 0.16 ± 0.02 | 0.16 ± 0.03 | 0.10 ± 0.01 |

## References

1. Clauset A, Shalizi CR, Newman MEJ. Power-law distributions in empirical data. SIAM Rev. 2009;51(4):661–703.
