# Supplementary material for: Coordinative structures as scale-free networks: Cascade and percolation dynamics in motor learning with empirical validation
Source: PLoS Comput Biol. 2026 Jul 21;22(7):e1014523. doi: 10.1371/journal.pcbi.1014523 (PMC13423191; doi:10.1371/journal.pcbi.1014523)
Supplement: S2 Appendix — Cascade propagation model derivations, stability function properties, and ensemble statistics. Tables A and B, Fig A. (DOCX) [file pcbi.1014523.s002.docx]

## S2 Appendix. Cascade Dynamics Model and Parameters

### Cascade dynamics model parameters

The cascade dynamics model (Eqs 2–4 in manuscript) implements a six-step update at each discrete timestep: (1) spontaneous potential generation (probability pₙ per node), (2) neighbor-mediated propagation (probability p_l per link from active nodes), (3) stability evaluation (Eq 2), (4) state transition (Eq 3), (5) coordination strength update, (6) cascade termination check.

**Table A.** Cascade dynamics model parameters.

| Parameter | Symbol | Value | Description |
| --- | --- | --- | --- |
| Spontaneous activation | pₙ | 0.1 | Per-timestep probability of gaining potential |
| Link propagation | p_l | 0.1 | Per-link transmission probability |
| Max stability | p_max | 0.99 | Upper bound on protection probability |
| Baseline stability | f₀ | 0.7 | Stability floor for all DOFs |
| Centrality weight | f₁ | 0.7 | Eigenvector centrality contribution |
| Reference constant | cₚ | 1.0 | Stability function scaling |
| Early-stage window | τ | 5 | Timesteps for R_early computation |

###

**Eigenvector centrality and the Perron–Frobenius basis**

Each degree of freedom is assigned an eigenvector centrality Cᵢ defined self-consistently as proportional to the summed centralities of its neighbors, λCᵢ = Σⱼ Aᵢⱼ Cⱼ, where A is the network adjacency matrix and λ is its largest eigenvalue; equivalently, the centrality vector is the leading eigenvector of A. For a connected, undirected network A is nonnegative and irreducible, so by the Perron–Frobenius theorem its largest eigenvalue is real, simple, and positive, and the associated eigenvector can be chosen with all entries strictly positive. The centrality Cᵢ is therefore uniquely defined and positive for every degree of freedom, with no sign ambiguity, and we normalize it so that Cᵢ ∈ (0, 1]. Because the measure weights each node by the centralities of its neighbors rather than by raw degree alone, hubs and the well-connected nodes adjacent to them receive the highest scores. This is the structural reason hub-initiated activation propagates across the network while peripheral activation remains localized (Table B), and the centrality-weighted stability fraction f_πi (Eq 4) inherits the same ordering, as detailed next.

### Stability function properties

The stability function π (Eq 2) has the following key properties: when f_π × s → 0, π → 0; when f_π × s → ∞, π → p_max; at f_π × s = cₚ, π = p_max/2. Since hub nodes have high Cᵢ, they have higher f_πi (Eq 4), producing greater stability and creating hierarchical recruitment: peripheral DOFs activate first (low stability), while hub DOFs activate later (high stability), ensuring that when hubs do activate, their high connectivity produces rapid system-wide propagation.

### Cascade ensemble statistics

Cascade measures: R_early = |A(τ=5)| / |A(0)| (propagation rate); R_∞ = |A(t→∞)| / N (cascade reach); Δ_hp = R_early(hub) / R_early(peripheral) (hub-peripheral asymmetry). Hub definition: degree > ⟨k⟩ + 2σ; peripheral: degree < ⟨k⟩.

**Table B.** Cascade dynamics ensemble statistics (100 realizations × 100 topology-isolated trials; pₙ = 0). Parameters from Table A.

| Measure | ER (Random) | WS (Small-World) | BA (Scale-Free) |
| --- | --- | --- | --- |
| R_early (hub) | 7.58 ± 0.94 | 4.98 ± 0.41 | 14.15 ± 1.97 |
| R_early (periph) | 3.41 ± 0.43 | 3.43 ± 0.28 | 3.99 ± 0.58 |
| R_∞ (hub) | 0.99 ± 0.01 | 0.99 ± 0.01 | 1.00 ± 0.00 |
| R_∞ (periph) | 0.90 ± 0.05 | 0.95 ± 0.03 | 0.91 ± 0.05 |
| Δ_hp | 2.25 ± 0.37 | 1.46 ± 0.18 | 3.60 ± 0.62 |

**Fig A. Cascade dynamics on a representative scale-free network.**

**
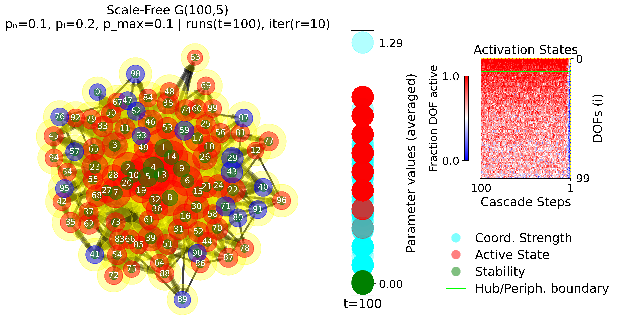
**

Note. A single realization of the cascade model (Eqs 2–4) on a BA network (N = 100, m = 5, seed = 42). The network (left) shows node states after the cascade: nodes that reached the active (failed) state in red, nodes remaining inactive in blue, and protected nodes as small green markers; node size is proportional to degree and arrows indicate the directed propagation paths. The scatter (center) reports the three state variables averaged across nodes at the final timestep: coordination strength, active state, and stability. The activation-state matrix (right) shows the fraction of each degree of freedom that is active across cascade steps, with degrees of freedom sorted by degree (hubs at top) and the green line marking the hub/peripheral boundary; the warmer band concentrated near the top illustrates the hub-first propagation that the differential Δ_hp quantifies in Table B. Parameters: pₙ = 0.1, pₗ = 0.2, p_max = 0.1, 100 timesteps, 10 realizations.
