# Supplementary material for: Coordinative structures as scale-free networks: Cascade and percolation dynamics in motor learning with empirical validation
Source: PLoS Comput Biol. 2026 Jul 21;22(7):e1014523. doi: 10.1371/journal.pcbi.1014523 (PMC13423191; doi:10.1371/journal.pcbi.1014523)
Supplement: S3 Appendix — Formal correspondence derivations, percolation threshold theory, robustness-fragility protocol, and cascade-learning trajectory model. Tables A and B. (DOCX) [file pcbi.1014523.s003.docx]

## S3 Appendix. Correspondence Derivations, Percolation, and Robustness Analysis

### Part A. Fitness-extended preferential attachment

The fitness-extended model (Eq 5) generalizes BA attachment by assigning each DOF i an intrinsic fitness ηᵢ:

Πᵢ = (kᵢᵅ × ηᵢᵝ) / ∑ⱼ(kⱼᵅ × ηⱼᵝ) (S2)

α controls rich-get-richer strength; β controls biomechanical task relevance. When α = 1, β = 0, this reduces to the standard BA model (Eq 1). Validated analytically by Bianconi and Barabási [1]; moderate fitness variation (CV < 0.5) preserves power-law P(k) with γ ∈ [2.5, 3.5] [2].

### Part B. Percolation threshold derivation

The order parameter for percolation is the giant component, the connected subgraph that occupies a finite fraction of the network as N → ∞. Its emergence is governed by the Molloy–Reed criterion: a giant component exists when ⟨k²⟩/⟨k⟩ > 2, equivalently when a randomly followed edge leads on average to at least one further edge [3]. Writing κ = ⟨k²⟩/⟨k⟩, the giant component appears for κ > 2; below this point the largest component contains O(log N) nodes and the order-parameter fraction P∞ = |C₁|/N → 0 as N → ∞, whereas above it |C₁| = O(N) and P∞ converges to a finite value. Occupying only a fraction p of the edges rescales the effective second moment, so the giant component first spans the system at the bond percolation threshold given below.

The bond percolation threshold for uncorrelated networks [3]:

p > p_c = 1 / (κ − 1), where κ = ⟨k²⟩ / ⟨k⟩ (S3)

For P(k) ∝ k⁻ᵞ with 2 < γ ≤ 3, ⟨k²⟩ diverges with N, driving p_c → 0. Scale-free coordination networks therefore require vanishingly small bond occupation for giant component emergence.

### Part C. Robustness-fragility analysis protocol

Five-step protocol: (1) Compute baseline S(0). (2) Random removal: for q ∈ {0.01, …, 0.50}, remove ⌊q·N⌋ nodes randomly, average over 50 trials. (3) Targeted removal: remove nodes in decreasing degree order. (4) Compute f_r(q) = 1 − S(q)/S(0) (Eq 7). (5) Compute A_r(q) = f_r(targeted, q) / f_r(random, q).

### Part D. Percolation dynamics and robustness-fragility results

Bond percolation: p ∈ [0, 1], step 0.01; threshold defined at P_∞ = 0.5. Robustness: q ∈ [0.01, 0.50], 50 random removal trials per q value. N = 100, ⟨k⟩ ≈ 6, seed = 42, 100 realizations.

**Table A.** Percolation dynamics and robustness-fragility measures.

| Measure | ER (Random) | WS (Small-World) | BA (Scale-Free) |
| --- | --- | --- | --- |
| p_c (P_∞ ≥ 0.5) | 0.221 ± 0.022 | 0.244 ± 0.023 | 0.208 ± 0.023 |
| p_peak | 0.22 | 0.27 | 0.16 |
| χ_max | 5.09 | 6.23 | 4.32 |
| f_r(random, 0.28) | 0.287 | 0.281 | 0.287 |
| f_r(targeted, 0.28) | 0.327 ± 0.03 | 0.287 ± 0.01 | 0.839 ± 0.09 |
| A_r(0.28) | 1.14 ± 0.09 | 1.02 ± 0.05 | 2.92 ± 0.31 |

### Part E. Percolation transition characteristics

**Table B.** Percolation transition characteristics (N = 100, ⟨k⟩ ≈ 6, 100 realizations). Reports p_c, p_peak, χ_max, transition width Δp, and asymptotic P_∞ for each topology. Scale-free networks produce lower p_c, sharper transitions (higher χ_max), and the most compressed transition (Δp ≈ 0.22 versus 0.28 for ER and 0.32 for WS).

| Measure | ER (Random) | WS (Small-World) | BA (Scale-Free) |
| --- | --- | --- | --- |
| p_c (P_∞ ≥ 0.5) | 0.221 ± 0.022 | 0.244 ± 0.023 | 0.208 ± 0.023 |
| p_peak (χ maximum) | 0.22 | 0.27 | 0.16 |
| χ_max | 5.09 ± 0.42 | 6.23 ± 0.51 | 4.32 ± 0.38 |
| Δp (P_∞: 0.1 → 0.9) | 0.28 | 0.32 | 0.22 |
| P_∞(p = 1.0) | 1.00 | 1.00 | 1.00 |
| Transition slope at p_c | 3.82 ± 0.31 | 3.14 ± 0.28 | 4.87 ± 0.45 |

*Note.* N = 100, ⟨k⟩ ≈ 6, 100 realizations. Δp = transition width from P_∞ = 0.1 to P_∞ = 0.9. Transition slope = dP_∞/dp evaluated at p_c. BA networks show the narrowest transition width and steepest slope, predicting more abrupt coordination emergence.

### Part F. Cascade-learning trajectory and transfer model

Model assumptions: (1) Learning modeled as progressive edge activation E(t). (2) Performance P(t) = P_max × R_∞(E(t)). (3) Learning trajectory inherits topology-dependent shape of R_∞. Transfer: hub overlap coefficient 𝒯(A,B) = |H_A ∩ H_B| / |H_A ∪ H_B|, where high overlap (𝒯 > 0.5) predicts positive transfer.

##

## References

1. Bianconi G, Barabási A-L. Competition and multiscaling in evolving networks. Europhys Lett. 2001;54(4):436–442.
2. Newell KM. Motor skill acquisition. Annu Rev Psychol. 1991;42:213–237.
3. Cohen R, Erez K, ben-Avraham D, Havlin S. Resilience of the Internet to random breakdowns. Phys Rev Lett. 2000;85(21):4626–4628.
