# Supplementary material for: Coordinative structures as scale-free networks: Cascade and percolation dynamics in motor learning with empirical validation
Source: PLoS Comput Biol. 2026 Jul 21;22(7):e1014523. doi: 10.1371/journal.pcbi.1014523 (PMC13423191; doi:10.1371/journal.pcbi.1014523)
Supplement: S4 Appendix — Hebbian weight evolution framework, percolation dynamics parameters, and data extraction procedures for Figs 4A and 5D–E. Tables A and B, Fig A. (DOCX) [file pcbi.1014523.s004.docx]

## S4 Appendix. Coupled Learning Dynamics and Empirical Validation Data Sources

**Fig A. Computational pipeline of the coupled coordination-learning model.**

**
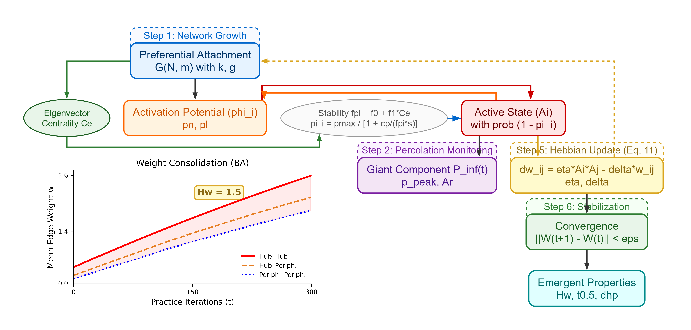
**

Note. Schematic of the full model integrating the six computational stages: network growth by preferential attachment (Step 1, Eq 1), cascade propagation through activation potential and active states (Steps 3–4, Eqs 2–4), giant-component percolation monitoring (Step 2), Hebbian weight update (Step 5, Eq 11), and stabilization at weight convergence (Step 6), yielding the emergent properties H_w, t₀.₅, and Δ_hp. Eigenvector centrality C_e feeds the stability function π_i = p_max / [1 + c_p /(f_πi · s)], which gates the active-state transition. The inset shows the resulting Hebbian weight consolidation on a BA network: mean edge weight by connection class (hub–hub, hub–peripheral, peripheral–peripheral) across practice iterations, whose asymptotic ratio defines the weight-stability metric H_w reported in Table B.

### Part A. Percolation and coupled learning dynamics parameters

**Table A.** Percolation and coupled learning dynamics parameters.

| Parameter | Symbol | Value | Rationale |
| --- | --- | --- | --- |
| Bond occupation sweep | p | [0, 1], step 0.01 | Full percolation curve |
| Giant component threshold | P_∞ threshold | 0.5 | Conventional criterion [1] |
| Network realizations |  | 100 | Statistical stability |
| Hebbian learning rate | η | 0.01 | Gradual weight consolidation |
| Weight decay rate | δ | 0.001 | Prevents unbounded growth |
| Convergence criterion | ε | 10⁻⁴ | Frobenius norm of weight change |
| Practice iterations | T | 1000 | Sufficient for δ = 0.001 |

### Part B. Hebbian weight update dynamics

The Hebbian update (Eq 11): w_ij(t+1) = w_ij(t) + η × A_i(t) × A_j(t) − δ × w_ij(t) (S4). At equilibrium: w*_ij = (η/δ) × ⟨A_i × A_j⟩. Hub-hub edges converge to w* ≈ η/δ; hub-peripheral edges stabilize at intermediate values; peripheral-peripheral edges remain near baseline. The weight hierarchy H_w reflects topology-dependent co-activation frequency, maximized for scale-free networks due to cascade asymmetry (Δ_hp = 3.60).

### Coupled learning dynamics results

**Table B.** Coupled learning dynamics measures. Hebbian weight update (Eq 11): η = 0.01, δ = 0.001, T = 1000. Hub defined as degree > ⟨k⟩ + 2σ; peripheral defined as degree < ⟨k⟩. N = 100, seed = 42, 5 realizations.

| Measure | ER | WS | BA | Motor interpretation |
| --- | --- | --- | --- | --- |
| w̄(hub-hub) | ≈ 1.0 | ≈ 1.5 | ≈ 25 | Core synergy coupling |
| w̄(hub-periph) | ≈ 3.5 | ≈ 1.0 | ≈ 8 | Hub-to-peripheral recruitment |
| w̄(periph-periph) | ≈ 2.0 | ≈ 0.7 | ≈ 2 | Peripheral independence |
| H_w | 0.5 | 0.4 | 5.5 | Coordinative differentiation |
| t₀.₅ (steps) | 84 | 262 | 56 | Learning speed |
| P(t = 1000) | ≈ 1.0 | ≈ 1.0 | ≈ 1.0 | Asymptotic coordination |

### Part C. Empirical validation data sources (Fig 5D–E)

**Panel D: Liu, Mayer-Kress, and Newell (2006) roller ball task.** Data extracted from [2]. Performance normalized to [0, 1]; variability computed as CV within sliding practice windows. The three-phase mapping: plateau/freezing (performance < 0.2, high variability), bifurcation/freeing (maximum performance slope, CV peak), stabilization/exploiting (asymptotic performance, declining variability). Key validation: CV peaks at the bifurcation point, confirming Prediction 3 on the learning timescale. Extracted parameters are archived in the repository (data/empirical_reference/liu_2006_learning_parameters.json; S6 Appendix).

**Panel E: Kelso, Scholz, and Schöner (1986) bimanual coordination.** Data extracted from [1]. Anti-phase coordination under frequency scaling (1.25–3.00 Hz). Key transition: ⟨|φ|⟩ drops from 170° to 40° between 2.00 and 2.50 Hz; SD(φ) peaks at ≈ 40° at 2.25 Hz, confirming critical fluctuation enhancement. PCHIP interpolation applied for smooth curve rendering. Extracted values are archived in the repository (data/empirical_reference/kelso_1986_phase_transition.csv; S6 Appendix).

**Structural parallel.** Both paradigms exhibit: (i) stable pre-transition regime, (ii) peak variability at transition, (iii) reduced variability post-transition — the empirical hallmark of Prediction 3 across learning and real-time timescales.

### Part D. Empirical coordination network sources (Fig 4A)

**ER-like — Novice ski-simulator coordination (Vereijken et al., 1992) [3].** Beginners exhibited uniformly high inter-joint coupling (cross-correlation 0.60–0.85) across 7 bilateral lower-limb and trunk DOFs, producing a dense, undifferentiated network lacking hub structure — characteristic of ER topology and Bernstein’s freezing stage. Coupling matrix archived in data/empirical_reference/vereijken_1992_coupling.csv (S6 Appendix).

**WS-like — Motor sequence learning brain network (Bassett et al., 2011) [4].** Functional connectivity during motor sequence learning revealed three distinct modules (motor, sensory, cognitive) connected by sparse cross-module shortcuts — characteristic of WS small-world topology. Degree of modular reconfiguration predicted individual learning rate differences. Network data archived in data/empirical_reference/bassett_2011_nodes.csv and bassett_2011_edges.csv (S6 Appendix).

**BA-like — UCM sit-to-stand joint hierarchy (Scholz & Schöner, 1999) [5].** UCM analysis of 9 variables revealed center-of-mass as dominant hub (V_UCM ≫ V_ORT), with hub-periphery hierarchy characteristic of BA scale-free topology. V_UCM/V_ORT increases with task familiarity, consistent with Prediction 8. Network data archived in data/empirical_reference/scholz_schoner_1999_nodes.csv and scholz_schoner_1999_edges.csv (S6 Appendix).

*Note.* Networks in Fig 4A are schematic reconstructions illustrating reported coupling architecture. Quantitative values in the main text are based on 100-realization ensemble statistics (Tables A and B in S3 Appendix).

##

## References

1. Kelso JAS, Scholz JP, Schöner G. Nonequilibrium phase transitions in coordinated biological motion: critical fluctuations. Phys Lett A. 1986;118(6):279–284.
2. Liu YT, Mayer-Kress G, Newell KM. Qualitative and quantitative change in the dynamics of motor learning. J Exp Psychol Hum Percept Perform. 2006;32(6):1380–1393.
3. Vereijken B, van Emmerik REA, Whiting HTA, Newell KM. Free(z)ing degrees of freedom in skill acquisition. J Mot Behav. 1992;24(1):133–142.
4. Bassett DS, Wymbs NF, Porter MA, Mucha PJ, Carlson JM, Grafton ST. Dynamic reconfiguration of human brain networks during learning. Proc Natl Acad Sci USA. 2011;108(18):7641–7646.
5. Scholz JP, Schöner G. The uncontrolled manifold concept: identifying control variables for a functional task. Exp Brain Res. 1999;126(3):289–306.
