# Supplementary material for: Coordinative structures as scale-free networks: Cascade and percolation dynamics in motor learning with empirical validation
Source: PLoS Comput Biol. 2026 Jul 21;22(7):e1014523. doi: 10.1371/journal.pcbi.1014523 (PMC13423191; doi:10.1371/journal.pcbi.1014523)
Supplement: S5 Appendix — Empirical grounding for eleven predictions with quantitative benchmarks, disconfirmation criteria, and suggested experimental protocols. Tables A and B. (DOCX) [file pcbi.1014523.s005.docx]

## S5 Appendix. Predicted Empirical Outcomes with Benchmarks

This appendix provides empirical grounding for the eleven predictions and five disconfirmation criteria (Tables 4 and S8; Fig 6).

**Perturbation propagation asymmetry (Predictions 1–2).** Ting and Macpherson [1] showed perturbation to hub muscles propagates across the full postural synergy while peripheral perturbation remains localized. Torres-Oviedo and Ting [2] extended this to standing postural responses. Chvatal and Ting [3] showed hub synergies generalize across balance and walking tasks.

**Three-phase sigmoid learning trajectory (Prediction 4).** Liu and Newell [4] provided direct evidence for S-shaped learning with plateaus of 3–17 days followed by jumps within 1–3 days. Newell, Liu, and Mayer-Kress [5] formalized nonlinear phase transitions in learning. Donner and Hardy [6] confirmed piecewise learning functions in a large-scale dataset (N > 10,000).

**Critical fluctuations and variability structure (Prediction 3).** Sternad [7] and Pacheco, Lafe, and Newell [8] showed variability structure changes qualitatively during learning transitions. Liu et al. [9] demonstrated CV peaks at the performance bifurcation (Fig 5D). Kelso et al. [10] showed SD peaks at the coordination phase transition (Fig 5E).

**Degree distribution maturation (Predictions 7–8).** d’Avella, Saltiel, and Bizzi [11] showed hub muscles contribute to multiple synergies with high loading weights. Boonstra et al. [12] constructed muscle networks from EMG coherence finding heterogeneous degree distributions in skilled postural control. Latash, Scholz, and Schöner [13] showed V_UCM/V_ORT increases with skill.

**Hub-synergy overlap (Prediction 9).** Tessari et al. [14] showed synergy expansion during skill acquisition follows a pattern consistent with preferential attachment to established hubs.

**Transfer magnitude vs. hub overlap (Predictions 10–11).** Cheung et al. [15] established synergy pairs with cosine similarity r > 0.7 predict behavioral generalization. Allen and Neptune [16] showed shared proximal modules enable locomotor transfer. Kang and Cauraugh [17] showed bilateral synergy overlap predicts force coordination recovery.

**Table A.** Additional predicted empirical outcomes with quantitative benchmarks (see Table 4 in main text for core predictions).

| # | Prediction | Model basis | Quantitative benchmark | Disconfirmation criterion |
| --- | --- | --- | --- | --- |
| 2 | Hub perturbation requires system-wide recovery | A_r = 2.92 (Eq 7) | Hub recovery 3–5× peripheral | Equivalent recovery times |
| 5 | Scale-free tasks learn faster | BA 33% faster than ER (Eq 11) | κ correlates with learning rate (r > 0.3) | No κ–learning speed correlation |
| 8 | Network matures with practice | Progressive strengthening (Eq 11) | κ increases monotonically | Static or decreasing κ |
| 9 | Hub DOFs = synergy leaders | Hub fraction ≈ 10% (Eq 1) | >60% overlap Cₑ hubs vs. PCA/NMF | Hubs uncorrelated with synergies |
| 11 | Asymmetric transfer: hub → broad benefit | R_∞(hub) = 1.00 (Eqs 2–4) | Whole-movement > part-practice | Part- and whole-practice equivalent |

*Note.* Predictions 2, 5, 8, 9, and 11 complement the six core predictions in Table 4. Each derives from simulation results mapped through the four correspondences (Table 1). Hub defined as degree > ⟨k⟩ + 2σ; peripheral defined as degree < ⟨k⟩.

**Statistical validation of core topological differences**

Statistical tests confirming the four principal differences between the scale-free (BA) topology and the random (ER) and small-world (WS) alternatives. Means and standard deviations are taken from the structural validation ensemble (Table D in S1 Appendix) and the percolation and robustness ensemble (Table A in S3 Appendix).

**Table B.** Statistical validation of the four core topological differences.

| Measure | Comparison | t | p (Bonferroni) | Cohen's d |
| --- | --- | --- | --- | --- |
| κ = ⟨k²⟩/⟨k⟩ (degree heterogeneity) | BA vs ER | 40.33 | <0.001 | 5.70 |
|  | BA vs WS | 60.64 | <0.001 | 8.58 |
| Gini (Cₑ) (hub concentration) | BA vs ER | 40.25 | <0.001 | 5.69 |
|  | BA vs WS | 46.08 | <0.001 | 6.52 |
| p_c (percolation threshold) | BA vs ER | −4.08 | <0.001 | −0.58 |
|  | BA vs WS | −11.07 | <0.001 | −1.57 |
| A_r (robustness asymmetry, q = 0.28) | BA vs ER | 55.14 | <0.001 | 7.80 |
|  | BA vs WS | 60.51 | <0.001 | 8.56 |

*Note.* Two-sided independent-samples t-tests with pooled variance, df = 198 (n = 100 realizations per topology). A positive value means BA is higher, a negative value means BA is lower. The negative signs for p_c confirm that BA has the lowest percolation threshold, as stated in the text. Cohen's d is the effect size, the size of the gap. All eight comparisons were significant after Bonferroni correction across topology pairs (α = 0.05, p < 0.001). Source means and standard deviations are in Table D in S1 Appendix and Table A in S3 Appendix.

## References

1. Ting LH, Macpherson JM. A limited set of muscle synergies for force control during a postural task. J Neurophysiol. 2005;93(1):609–613.
2. Torres-Oviedo G, Ting LH. Muscle synergies characterizing human postural responses. J Neurophysiol. 2007;98(4):2144–2156.
3. Chvatal SA, Ting LH. Common muscle synergies for balance and walking. Front Comput Neurosci. 2013;7:48.
4. Liu YT, Newell KM. S-shaped motor learning and nonequilibrium phase transitions. J Exp Psychol Hum Percept Perform. 2015;41(2):403–414.
5. Newell KM, Liu YT, Mayer-Kress G. Time scales in motor learning and development. Psychol Rev. 2001;108(1):57–82.
6. Donner Y, Hardy JL. Piecewise power laws in individual learning curves. Psychon Bull Rev. 2015;22(5):1308–1319.
7. Sternad D. It’s not (only) the mean that matters: variability, noise and exploration in skill learning. Curr Opin Behav Sci. 2018;20:183–195.
8. Pacheco MM, Lafe CW, Newell KM. Search strategies in the perceptual-motor workspace. Front Psychol. 2019;10:1874.
9. Liu YT, Mayer-Kress G, Newell KM. Qualitative and quantitative change in the dynamics of motor learning. J Exp Psychol Hum Percept Perform. 2006;32(6):1380–1393.
10. Kelso JAS, Scholz JP, Schöner G. Nonequilibrium phase transitions in coordinated biological motion: critical fluctuations. Phys Lett A. 1986;118(6):279–284.
11. d’Avella A, Saltiel P, Bizzi E. Combinations of muscle synergies in the construction of a natural motor behavior. Nat Neurosci. 2003;6(3):300–308.
12. Boonstra TW, Danna-Dos-Santos A, Xie HB, Roerdink M, Stins JF, Breakspear M. Muscle networks: connectivity analysis of EMG activity during postural control. Sci Rep. 2015;5:17830.
13. Latash ML, Scholz JP, Schöner G. Motor control strategies revealed in the structure of motor variability. Exerc Sport Sci Rev. 2002;30(1):26–31.
14. Tessari A, Chisari C, Taborri J, De Marchis C. Explaining human motor coordination via the synergy expansion hypothesis. Proc Natl Acad Sci USA. 2025;122(13):e2501705122.
15. Cheung VCK, d’Avella A, Tresch MC, Bizzi E. Central and sensory contributions to the activation and organization of muscle synergies. J Neurosci. 2005;25(27):6419–6434.
16. Allen JL, Neptune RR. Three-dimensional modular control of human walking. J Biomech. 2012;45(12):2157–2163.
17. Kang N, Cauraugh JH. Bilateral synergy as an index of force coordination in chronic stroke. Exp Brain Res. 2014;232(12):4211–4221.
