# Supplementary material for: Coordinative structures as scale-free networks: Cascade and percolation dynamics in motor learning with empirical validation
Source: PLoS Comput Biol. 2026 Jul 21;22(7):e1014523. doi: 10.1371/journal.pcbi.1014523 (PMC13423191; doi:10.1371/journal.pcbi.1014523)
Supplement: S6 Appendix — Repository structure, simulation scripts, empirical reference data, and reproduction instructions. (DOCX) [file pcbi.1014523.s006.docx]

## S6 Appendix. Open-Source Code Repository and Reproduction Guide

All simulation code, empirical reference data, and reproduction instructions for this study are publicly available at:

https://github.com/pcw8531/Coordinative-structures-scale-free-networks

The repository is licensed under MIT and is archived on Zenodo with the persistent DOI 10.5281/zenodo.20694466.

### Repository structure

The repository contains two primary components: (1) simulation scripts implementing all computational models described in Sections 2.1–2.4 of the manuscript, and (2) empirical reference data files used for the validation analyses in Figures 4–5.

### Simulation scripts

**01_network_generation.py** (Section 2.1, Eq 1). Generates ER, WS, and BA network ensembles (N = 100, ⟨k⟩ ≈ 6, 100 realizations, seed = 42) and computes structural metrics: κ, γ̂, Gini coefficient, hub fraction, and KS goodness-of-fit. Reproduces Tables A–D in S1 Appendix and Table 3 (structural rows).

**02_cascade_dynamics.py** (Section 2.2, Eqs 2–4). Implements the six-step cascade update cycle with parameters from Table A in S2 Appendix. Computes R_early, R∞, and Δ_hp for hub-initiated and peripheral-initiated cascades. Reproduces Tables A and B in S2 Appendix, and Table 3 (cascade rows).

**03_percolation_robustness.py** (Sections 2.3–2.4, Eqs 6–7, 9–10). Performs bond percolation sweeps (p ∈ [0, 1], step 0.01) computing P∞(p) and χ(p), and robustness-fragility analysis via random and targeted node removal. Reproduces Tables A and B in S3 Appendix, and Table 3 (percolation rows).

**04_coupled_learning.py** (Section 2.4, Eqs 5, 11–12). Implements the coupled learning algorithm with Hebbian weight updates (η = 0.01, δ = 0.001, T = 1000). Computes performance trajectories P∞(t), weight hierarchy H_w, and learning half-time t₀.₅. Reproduces Tables A and B in S4 Appendix, and Table 3 (learning rows).

Each script includes automated verification, printing simulated values alongside manuscript table values with tolerance checks. All scripts share identical base parameters (N = 100, seed = 42, ⟨k⟩ ≈ 6) ensuring cross-script consistency.

### Empirical reference data

The data/empirical_reference/ directory contains network-reinterpreted representations of empirical findings from five published studies used for model validation in Figures 4–5. All values are directly traceable to the original publications and verified against the original publications.

*Fig 4A sources:* vereijken_1992_coupling.csv (7-DOF inter-joint coupling matrix, ER-like topology); bassett_2011_nodes.csv and bassett_2011_edges.csv (12-region brain network, WS-like topology); scholz_schoner_1999_nodes.csv and scholz_schoner_1999_edges.csv (9-variable UCM hierarchy, BA-like topology).

*Fig 5D–E sources:* liu_2006_learning_parameters.json (roller ball task performance and variability parameters); kelso_1986_phase_transition.csv (bimanual coordination phase transition data at 8 driving frequencies).

Complete provenance documentation is provided in data/empirical_reference/README.md within the repository.

### Reproduction instructions

To reproduce all computational results: (1) clone the repository; (2) install dependencies via pip install -r requirements.txt (Python ≥ 3.9, NetworkX, NumPy, SciPy, Matplotlib); (3) execute the four scripts sequentially (01 through 04). Each script generates CSV and NPZ output files in data/simulation_outputs/ and prints verification summaries to the console. The complete reproduction pipeline requires approximately 30–60 minutes on a standard desktop workstation.

## 
