# Supplementary material for: Coordinative structures as scale-free networks: Cascade and percolation dynamics in motor learning with empirical validation
Source: PLoS Comput Biol. 2026 Jul 21;22(7):e1014523. doi: 10.1371/journal.pcbi.1014523 (PMC13423191; doi:10.1371/journal.pcbi.1014523)
Supplement: Attachment — Submitted filename: main.pdf [file pcbi.1014523.s007.pdf]

The article primarily presents a mapping between 'Coordinative Structures', a term used for self-emerging functional groupings of degrees of freedom (DOF) in Generalized Muscular Programs (GMPs) and 'Scale-Free networks', a graph structure with a power-law degree distribution as proposed by Barabási and Albert. The author first describes 'Bernstein's problem', the theoretical framework for the self-emergent functional grouping of human muscles to achieve locomotion. Next, the author introduces relevant concepts from network science and metrics such as graph generation algorithms for ER (Random), WS (Small-world), and BA (Scale-free) networks, percolation threshold, cascading dynamics, and others, which are used throughout the manuscript. Four formal conjectures are then proposed to provide a mapping between network topologies and the Coordinative Structure. Furthermore, the predictions from these conjectures are demonstrated both numerically and with the help of empirical datasets. Finally, the author discusses the implications of the results, practical implementations, their relation to prior work and limitations, and future avenues to explore within this framework.

The investigation is exhaustive in its coverage of the different aspects of muscular synergies (Coordinative Structures) and network topologies. The article title accurately reflects the investigation presented. The author has taken particular care to explain all relevant terminology in a contextual fashion and has provided an extensive appendix that goes into depth regarding the metrics. The author has compared four different network topologies and presented formal predictions, supported by statistical tests, to support the mapping. The author has done a great job in explaining the details of each method, the methodology, and the outputs.

I also wish to highlight the author's effort in providing both theoretical and practical implications (particularly regarding training athletes to improve muscle coordination), as well as their consideration on discussion on the study's limitations. The author has made a significant effort in presenting and supporting his conjectures, which is valuable for advancing the field. I see tremendous value in this work and believe it addresses an important aspect of the current literature. Therefore, I recommend this article for publication but with some minor revisions. I list some of my comments below, which I hope the author will take into consideration before submitting a revision.

<u>Comments:</u> A general technical comment would be to add section and subsection numbers to various paragraphs throughout the manuscript, and use references to link previous metric explanations to the results and discussions.

1. line 126; page-3. In the introduction of percolation theory and phase transitions in networks, the concept of giant connected components is introduced without further explanation and only a few references are provided. A brief mathematical introduction of the giant component would be helpful here, as it is central to defining percolation thresholds.

2. line 137; page-4. Similarly, Small World Networks are introduced here without a brief explanation of the WS algorithm, in contrast to ER or BA, which are briefly described.

3. Line 244/249; page-6/7 A visual distinction or a small note would be helpful here to distinguish between eigenvector centrality components and the reference constant. In particular,

I believe the concept of eigenvector centrality, the role of this metric in cascades in a network, and the relevance of Perron-Frobenius theory should be briefly explained to help readers understand cascades better.

4. Line 269 (Table 1, col 3, row 2); page-7 In the 'Formal Mechanism' column, '... Fitness-extended PA kernel', I believe it should be BA kernel.

5. Line 323; page-9 $\mathcal{C}_1(p)$ should be clearly described as the principal centrality factor indicating the giant component of the network.

6. Table 2; Line 392; page-11 It would be helpful to refer back to the metric explanations in previous sections for this column.

7. Fig.2; page-11 It would be helpful to have the histogram on the Y axis with bars on the X axis to better visualize the scatter plot shown in the inset of the figure.

8. Fig.7; page-21 The ternary figures should be clearly described, including what is being shown and the intended results.

Overall, I feel the investigation is worthwhile and would like to thank the author for their efforts in writing this manuscript. I hope the author will take my comments into consideration and further improve their work in the future. I recommend this research for publication with minor revisions.
