# Supplementary material for: Coordinative structures as scale-free networks: Cascade and percolation dynamics in motor learning with empirical validation
Source: PLoS Comput Biol. 2026 Jul 21;22(7):e1014523. doi: 10.1371/journal.pcbi.1014523 (PMC13423191; doi:10.1371/journal.pcbi.1014523)
Supplement: Attachment — Submitted filename: Response to Reviewers.docx [file pcbi.1014523.s009.docx]

**Point-by-Point Response to Editor and Reviewers**

Title: Coordinative structures as scale-free networks: cascade and percolation dynamics in motor learning with empirical validation

Manuscript ID: PCOMPBIOL-D-26-00505	|	PLOS Computational Biology	|	June 2026

Chulwook Park, Department of Physical Education, Seoul National University

Dear Dr. Shinde, Dr. Birtwistle, and Reviewers,

We sincerely thank Guest Editor Dr. Pramod Shinde, Section Editor Dr. Marc Birtwistle, and both reviewers for the constructive and encouraging assessment, and for the recommendation to publish after revision. We have addressed every point through revisions to the manuscript and the Supporting Information. Below, each original comment is quoted in italics, followed by our response and a description of the specific changes made. All revisions are recorded as tracked changes and additionally highlighted in red in the marked-up manuscript so that each revised passage can be located at a glance.

**Response to the Editor**

**Editor:** *“My decision is major revision. Neither reviewer requests new experiments. Their concerns center on clarity, justification, and overstatement of claims, and I am confident you can resolve them. Please address every point in both appended reports.”*

**Response:** We have addressed every point from both reports, as detailed in the Reviewer sections below. We respond first to the three themes the Editor highlighted, then to the specific items and to the calibration of claims.

**Theme 1: Justify the key metrics**

**Comment:** *“First, justify your key metrics. Explain why you chose degree heterogeneity and the eigenvector centrality Gini coefficient, state what each one represents for coordinative structures, and show how they support your claims. Add the short conceptual explanations the reviewers ask for so the paper stands on its own.”*

**Response:** We have added explicit justifications for both metrics in the Materials and Methods. In Network Model Specifications (Section 2.1), we now explain that, because all three topologies are matched on mean degree, degree heterogeneity κ = ⟨k²⟩/⟨k⟩ is the quantity that distinguishes a hub-organized network from a homogeneous one. We further state that κ is not an arbitrary score but the structural quantity that governs the dynamics, since it sets the percolation threshold (p_c = 1/(κ − 1)) and diverges with network size for scale-free distributions, and that a higher κ in the scale-free model is meaningful because it reflects connectivity concentrated in a few hub degrees of freedom, the substrate for the leading-element hierarchy. We also explain that the eigenvector-centrality Gini quantifies inequality of propagated influence, testing the same hierarchy property as κ from a complementary dynamical perspective. The conceptual explanation of eigenvector centrality is provided in Cascade Dynamics Model (Section 2.2).

**Changes:** Rationale for κ added in Section 2.1; rationale and interpretation of the eigenvector-centrality Gini added in Sections 2.1 and 2.2. This theme is shared with Reviewer 2, Point 1, and Reviewer 1, Point 3.

**Theme 2: Define the target properties up front and add a summary table**

**Comment:** *“Second, define your target properties up front and add a summary table. State the characteristic properties of coordinative structures before the Results, and include a table that links each property to its network measurement, the reason for that measurement, and the statistic you compute.”*

**Response:** We have added a new subsection, Target properties of coordinative structures and their network signatures (Section 2.6), at the end of the Materials and Methods, immediately before Computational Implementation and Statistical Analysis and therefore before the Results. It names the five properties the model must reproduce and states the explicit criterion by which each is judged reproduced: presence in the scale-free topology and statistical separation from the random and small-world baselines. We also added a new summary table (Table 2) linking each property to its network measurement, the rationale, and the statistic computed, with symbol definitions in the table note.

**Changes:** New Section 2.6 added before the Results; new Table 2 added. The former Tables 2 and 3 are renumbered Tables 3 and 4, and all in-text and Supporting Information cross-references are updated. This theme is shared with Reviewer 2, Point 2.

**Theme 3: Improve the readability of the Results**

**Comment:** *“Third, improve the readability of the Results. Both reviewers found this section hard to follow, so rewrite it for clarity, number your sections, and cross-reference your earlier metric definitions where you report results.”*

**Response:** We improved the Results in three ways without altering any reported value. First, we added section and subsection numbering throughout the manuscript (Sections 1 to 4). Second, we added a short preamble to Section 3 stating that the subsections test, in turn, the five target properties defined in Section 2.6, and we opened each Results subsection (3.1 to 3.5) with a sentence naming the property it tests and the Methods section where the relevant measures are defined. Third, we inserted cross-references at the first mention of each metric in the Results, pointing back to its definition in the Materials and Methods.

**Changes:** Section and subsection numbering added throughout; Results preamble added; each Results subsection opens with the property under test and a Methods cross-reference. This theme is shared with Reviewer 2, Point 3, and Reviewer 1’s general comment.

**Specific line, figure, and table items (including the Table 1 typo)**

**Comment:** *“Please also fix the specific line, figure, and table items listed in the appended reports, including the typo in Table 1.”*

**Response:** Each specific item is addressed individually under the corresponding reviewer point below. The Table 1 typo (Reviewer 1, Point 4) now reads “Fitness-extended BA kernel with percolation threshold.” The two figure refinements, Fig 2 (Reviewer 1, Point 7) and Fig 7 (Reviewer 1, Point 8), are complete and incorporated in the revised figure files.

**Changes:** Table 1 corrected; Fig 2 and Fig 7 revised. See Reviewer 1, Points 4, 7, and 8.

**Calibration of claims**

**Comment:** *“Their concerns center on clarity, justification, and overstatement of claims.”*

**Response:** We recalibrated our claims so that every statement is bounded to what the three-topology comparison supports. In the Abstract, the statement that scale-free topology uniquely reproduces coordinative structure properties now reads that it reproduces them more completely than the random and small-world alternatives. In the Introduction (Sections 1.2 and 1.3), claims that scale-free topology is specifically required are reframed as comparative claims that it best reproduces, or reproduces more completely than, the alternatives. In the Results, three exclusivity phrases are bounded to the three topologies tested: the cascade signature is identified as distinguishing the scale-free model rather than establishing a universal signature (Section 3.2); the robust-yet-fragile signature is described as appearing only in the scale-free model among the three topologies (Section 3.3); and coordinative differentiation is described as emerging only in the scale-free model among the three compared (Section 3.4).

**Changes:** Abstract, Introduction (Sections 1.2 and 1.3), and Results (Sections 3.2, 3.3, 3.4) reworded. No reported value, statistic, or figure was changed; only the strength of the inferential framing was adjusted.

**Response to Reviewer 1**

We thank Reviewer 1 for the careful reading and the supportive recommendation, and for noting “tremendous value in this work.” We respond to the general comment and each numbered point below.

**General comment**

**Comment:** *“A general technical comment would be to add section and subsection numbers to various paragraphs throughout the manuscript, and use references to link previous metric explanations to the results and discussions.”*

**Response:** We have added section and subsection numbering throughout the manuscript (Sections 1 to 4, with subsections such as 2.1 to 2.7 and 3.1 to 3.5). We also added cross-references linking the metric definitions in the Materials and Methods to the points where those metrics are reported in the Results, both at first mention in each subsection and in the note to the consolidated results table (Table 3; see Point 6).

**Changes:** Numbering added throughout; metric cross-references added in the Results and in the Table 3 note.

**Point 1: Giant connected component**

**Comment:** *“line 126; page-3. In the introduction of percolation theory and phase transitions in networks, the concept of giant connected components is introduced without further explanation and only a few references are provided. A brief mathematical introduction of the giant component would be helpful here, as it is central to defining percolation thresholds.”*

**Response:** We have added a brief mathematical description of the giant connected component in Section 1.2, where percolation and phase transitions are introduced. It states that the giant component is the connected subgraph whose size scales with the number of nodes N: below threshold every component contains O(log N) nodes and occupies a vanishing fraction of the network, whereas above threshold a single component contains O(N) nodes and occupies a finite fraction, and that the emergence of this extensive component at p_c is the structural event that defines the percolation transition.

**Changes:** Mathematical definition of the giant component added in Section 1.2; a fuller derivation is provided in S3 Appendix.

**Point 2: Watts–Strogatz construction**

**Comment:** *“line 137; page-4. Similarly, Small World Networks are introduced here without a brief explanation of the WS algorithm, in contrast to ER or BA, which are briefly described.”*

**Response:** We have added a brief description of the Watts–Strogatz construction at the point in Section 1.2 where the three topologies are compared, so that WS is now described by its generating mechanism, the rewiring of a fraction of the edges of a regular ring lattice, in parallel with the random and scale-free models. The full WS algorithm, including the rewiring probability β, was already specified in Section 2.1, which the introductory mention now complements.

**Changes:** WS construction added in Section 1.2; full algorithm retained in Section 2.1.

**Point 3: Eigenvector centrality and Perron–Frobenius theory**

**Comment:** *“Line 244/249; page-6/7 A visual distinction or a small note would be helpful here to distinguish between eigenvector centrality components and the reference constant. In particular, I believe the concept of eigenvector centrality, the role of this metric in cascades in a network, and the relevance of Perron-Frobenius theory should be briefly explained to help readers understand cascades better.”*

**Response:** We now explain eigenvector centrality, its role in cascades, and the Perron–Frobenius basis in Cascade Dynamics Model (Section 2.2). Each node’s centrality Cᵢ is the corresponding entry of the leading eigenvector of the adjacency matrix; the Perron–Frobenius theorem guarantees this eigenvector is unique and strictly positive for a connected nonnegative network, so Cᵢ is well defined and positive for every degree of freedom; and high-centrality hubs therefore propagate activation rapidly while peripheral nodes remain localized, which is the dynamical origin of the hub-peripheral asymmetry Δ_hp. We also added a note distinguishing the centrality terms (Cᵢ, the node-specific eigenvector centrality, and f_πi, the centrality-weighted stability fraction) from the global reference constant c_p.

**Changes:** Eigenvector-centrality and Perron–Frobenius explanation and the Cᵢ versus c_p note added in Section 2.2; a fuller treatment is provided in S2 Appendix.

**Point 4: Table 1 typo**

**Comment:** *“Line 269 (Table 1, col 3, row 2); page-7 In the ’Formal Mechanism’ column, ‘... Fitness-extended PA kernel’, I believe it should be BA kernel.”*

**Response:** Corrected. In the Formal Mechanism column of Table 1, the entry now reads “Fitness-extended BA kernel with percolation threshold.”

**Changes:** Table 1, Formal Mechanism column, corrected from “PA kernel” to “BA kernel.”

**Point 5: Description of C₁(p)**

**Comment:** *“Line 323; page-9 C1(p) should be clearly described as the principal centrality factor indicating the giant component of the network.”*

**Response:** We have added a sentence immediately after Eq 9 (Section 2.4) stating that C₁(p) denotes the largest connected component (the giant component) of the network at bond-occupation level p, with N the total number of nodes, so that the order parameter p∞ is the fraction of nodes belonging to the giant component and C₁(p) is the structural quantity whose emergence marks the percolation transition. This is consistent with the giant-component definition added in Section 1.2 (Point 1).

**Changes:** Description of C₁(p) added after Eq 9 in Section 2.4.

**Point 6: Refer back to metric definitions in the results table**

**Comment:** *“Table 2; Line 392; page-11 It would be helpful to refer back to the metric explanations in previous sections for this column.”*

**Response:** We have added to the note of the consolidated results table the Materials and Methods section where each measure is defined: κ and γ̂ in Section 2.1, Δ_hp in Section 2.2, A_r in Section 2.3, and p_peak, H_W, and t_0.5 in Section 2.4. With the new summary table added under Editor Theme 2, this consolidated results table is now numbered Table 3.

**Changes:** Per-measure Methods cross-references added to the Table 3 note.

**Point 7: Fig 2 inset visualization**

**Comment:** *“Fig.2; page-11 It would be helpful to have the histogram on the Y axis with bars on the X axis to better visualize the scatter plot shown in the inset of the figure.”*

**Response:** Following the reviewer’s suggestion, we revised Fig 2. The pooled eigenvector-centrality distribution is now displayed as a relative-frequency histogram, with relative frequency on the vertical axis and the bars positioned along the horizontal centrality axis, overlaid with a dotted kernel-density curve. The inset is now a box-and-scatter plot in which the 100 per-realization κ values are shown as jittered points behind the box, directly addressing the request to better visualize the scatter.

**Changes:** Fig 2 regenerated and its caption updated to describe the relative-frequency histogram and the box-and-scatter κ inset.

**Point 8: Description of the Fig 7 ternary plots**

**Comment:** *“Fig.7; page-21 The ternary figures should be clearly described, including what is being shown and the intended results.”*

**Response:** We expanded the Fig 7 caption to describe the ternary state-space plots explicitly. The caption now states that each simplex is spanned by the three normalized coordination signatures, coordination (giant-component fraction p∞), cascade spread (Δ_hp), and weight stability (H_W), scaled so the three coordinates sum to one; that each point is the network’s coordination state at one learning stage; and that the trajectory moves toward the high-p∞ region as coordination develops. The upper layer presents this as five sequential ternary plots across practice, and the bottom layer situates the same state space in the central ternary inset of the learning-trajectory panel. To keep the main figure focused, the algorithmic flowchart and the single-realization cascade prototype have been moved to the Supporting Information (S2 Fig and S1 Fig, respectively).

**Changes:** Fig 7 caption expanded; the algorithmic flowchart and the single-realization prototype relocated to S2 Fig and S1 Fig.

**Remarks.** We thank Reviewer 1 for these constructive and detailed comments. The added explanations of the giant component, the Watts–Strogatz construction, eigenvector centrality and Perron–Frobenius theory, together with the corrected Table 1 entry and the improved Fig 2 and Fig 7 presentations, have strengthened the clarity and self-containment of the manuscript.

**Response to Reviewer 2**

We thank Reviewer 2 for the positive assessment, describing the study as “an insightful study that explores the connection between scale-free networks and coordinative structures,” and for the suggestions that improve the paper’s logical flow and readability. We respond to each point below.

**Point 1: Justification of the metrics**

**Comment:** *“In the Results section, several methodological choices are not sufficiently explained. For example, it is unclear why degree heterogeneity is selected as a key measurement. Why does a higher degree heterogeneity in the BA model indicate a better or more meaningful result? Similarly, the interpretation of the eigenvector centrality Gini coefficient is not well articulated. The authors should clarify what these metrics represent in the context of coordinative structures and how they support the main claims.”*

**Response:** We have added the requested explanations to the Materials and Methods. We now state why degree heterogeneity κ is a key measurement (it is the only structural quantity that separates the three mean-degree-matched topologies and it governs the network’s percolation dynamics), why a higher κ in the scale-free model is the meaningful result rather than an arbitrary larger value (it reflects the hub-periphery concentration that defines a coordinative hierarchy), and what the eigenvector-centrality Gini represents (inequality of dynamical influence, identifying a few leading degrees of freedom that coordinate many subordinate ones). These definitions are cross-referenced from the Results where the metrics are reported.

**Changes:** Rationale and interpretation for κ and the eigenvector-centrality Gini added in Sections 2.1 and 2.2, with cross-references from the Results. This point is shared with Editor Theme 1 and Reviewer 1, Point 3.

**Point 2: Define the properties up front and add a summary table**

**Comment:** *“Since the goal is to establish a connection between scale-free networks and the characteristic properties of coordinative structures, these properties should be explicitly defined before presenting the experimental results. It would be helpful to include a summary table that outlines: (i) the specific properties of interest, (ii) the corresponding network measurements used to capture each property, (iii) the rationale for selecting these measurements, and (iv) what is the statistics calculated in the experiment. This may improve the logical flow of the paper.”*

**Response:** We have implemented this suggestion in full. A new subsection at the end of the Materials and Methods (Section 2.6), before the Results, defines the five characteristic properties, and a new summary table (Table 2) provides exactly the requested columns: (i) the property, (ii) the network measurement capturing it, (iii) the rationale, and (iv) the statistic computed. We are grateful for this suggestion, which improves the logical flow as the reviewer anticipated.

**Changes:** New Section 2.6 and new Table 2 added before the Results. See also Editor Theme 2.

**Point 3: Readability of the Results**

**Comment:** *“The topic is interesting, and the simulation model appears reasonable. However, the manuscript needs to improve its readability. The current description in the Results section makes it difficult to grasp the main idea.”*

**Response:** We have made the Results easier to follow by numbering the sections, adding a preamble to Section 3 that frames the subsections as tests of the five target properties defined in Section 2.6, and opening each subsection with a sentence stating the property under test and its headline result. Together with the up-front property definitions (Point 2) and the metric justifications (Point 1), the central idea of each subsection is now signposted.

**Changes:** Section numbering, Results preamble, and per-subsection framing added. This point is shared with Editor Theme 3.

**Remarks.** We thank Reviewer 2 for the rigorous and constructive evaluation. The up-front definition of the target properties, the new summary table, the metric justifications, and the improved Results structure have together clarified the logical flow and the central message of the paper.

**Journal Requirements**

Per the decision letter, the technical and formatting items listed under Journal Requirements are addressed within the Editorial Manager submission form. For completeness, these comprise the main figures supplied as separate .tif files validated with the NAAS tool; the Financial Disclosure and Funding Information entries, with the funders and grant numbers matched in the same order across both fields; the Competing Interests statement; the Data Availability statement, with the repository archived on Zenodo under the persistent DOI 10.5281/zenodo.20694466; and ORCID verification. These are completed at resubmission.

**Closing Remarks**

We believe these revisions address all of the Editor’s and reviewers’ comments and have strengthened the clarity, justification, and rigor of the manuscript. We thank the Editor and reviewers again for their time and constructive guidance.

Respectfully submitted,

**Chulwook Park**

BK21 Associate Professor, Department of Physical Education

Seoul National University, Seoul 08826, South Korea

IIASA, Laxenburg, Austria | OIST, Okinawa, Japan

pcw8531@snu.ac.kr | ORCiD: 0000-0001-8714-5760
