# Supplementary material for: Coordinative structures as scale-free networks: Cascade and percolation dynamics in motor learning with empirical validation
Source: PLoS Comput Biol. 2026 Jul 21;22(7):e1014523. doi: 10.1371/journal.pcbi.1014523 (PMC13423191; doi:10.1371/journal.pcbi.1014523)
Supplement: Peer review history

# Peer review: Decision Letter 0

**DOI:** 10.1371/journal.pcbi.1014523.r001

**Authors:** Marc R Birtwistle, Pramod Shinde

9 Jun 2026

PCOMPBIOL-D-26-00505

Coordinative structures as scale-free networks: cascade and percolation dynamics in motor learning with empirical validation

PLOS Computational Biology

Dear Dr. Park,

Thank you for submitting your manuscript to PLOS Computational Biology. After careful consideration, we feel that it has merit but does not fully meet PLOS Computational Biology's publication criteria as it currently stands. Therefore, we invite you to submit a revised version of the manuscript that addresses the points raised during the review process.

Please submit your revised manuscript by Aug 09 2026 11:59PM. If you will need more time than this to complete your revisions, please reply to this message or contact the journal office at ploscompbiol@plos.org. When you're ready to submit your revision, log on to https://www.editorialmanager.com/pcompbiol/ and select the 'Submissions Needing Revision' folder to locate your manuscript file.

Please include the following items when submitting your revised manuscript:

* A letter that responds to each point raised by the editor and reviewer(s). You should upload this letter as a separate file labeled 'Response to Reviewers'. This file does not need to include responses to formatting updates and technical items listed in the 'Journal Requirements' section below.

* A marked-up copy of your manuscript that highlights changes made to the original version. You should upload this as a separate file labeled 'Revised Manuscript with Track Changes'.

* An unmarked version of your revised paper without tracked changes. You should upload this as a separate file labeled 'Manuscript'.

If you would like to make changes to your financial disclosure, competing interests statement, or data availability statement, please make these updates within the submission form at the time of resubmission. Guidelines for resubmitting your figure files are available below the reviewer comments at the end of this letter. As the corresponding author, your ORCID iD is verified in the submission system and will appear in the published article. PLOS supports the use of ORCID, and we encourage all coauthors to register for an ORCID iD and use it as well. Please encourage your coauthors to verify their ORCID iD within the submission system before final acceptance, as unverified ORCID iDs will not appear in the published article. Only the individual author can complete the verification step; PLOS staff cannot verify ORCID iDs on behalf of authors.

We look forward to receiving your revised manuscript.

Kind regards,

Pramod Shinde

Guest Editor

PLOS Computational Biology

Marc Birtwistle

Section Editor

PLOS Computational Biology

**Additional Editor Comments (if provided):**

Thank you for submitting your manuscript to PLOS Computational Biology. Two reviewers have assessed it, and both recommend publication after revision. Their full reports are appended with this letter.

My decision is major revision. Neither reviewer requests new experiments. Their concerns center on clarity, justification, and overstatement of claims, and I am confident you can resolve them.

Please address every point in both appended reports. Three themes run through both reviews, and I ask you to give them particular attention. First, justify your key metrics. Explain why you chose degree heterogeneity and the eigenvector centrality Gini coefficient, state what each one represents for coordinative structures, and show how they support your claims. Add the short conceptual explanations the reviewers ask for so the paper stands on its own. Second, define your target properties up front and add a summary table. State the characteristic properties of coordinative structures before the Results, and include a table that links each property to its network measurement, the reason for that measurement, and the statistic you compute. Third, improve the readability of the Results. Both reviewers found this section hard to follow, so rewrite it for clarity, number your sections, and cross-reference your earlier metric definitions where you report results.

Please also fix the specific line, figure, and table items listed in the appended reports, including the typo in Table 1.

Return your revised manuscript with a point-by-point response to each reviewer comment. State clearly what you changed and where.

I look forward to your revision.

**Journal Requirements:**

If the reviewer comments include a recommendation to cite specific previously published works, please review and evaluate these publications to determine whether they are relevant and should be cited. There is no requirement to cite these works unless the editor has indicated otherwise.

1) Please upload all main figures as separate Figure files in .tif or .eps format. For more information about how to convert and format your figure files please see our guidelines:

https://journals.plos.org/ploscompbiol/s/figures

2) Please upload a copy of Figure Fig 1-7 which you refer to in your text on pages 4, 11, 12, 13, 15, and 17. Or, if the figure is no longer to be included as part of the submission please remove all reference to it within the text.

3) Thank you for stating "The repository will be archived on Zenodo with a persistent DOI upon acceptance.. We strongly recommend all authors deposit their data before acceptance, as the process can be lengthy and hold up publication timelines. Please note that, though access restrictions are acceptable now, your entire minimal dataset will need to be made freely accessible if your manuscript is accepted for publication. This policy applies to all data except where public deposition would breach compliance with the protocol approved by your research ethics board. If you are unable to adhere to our open data policy, please kindly revise your statement to explain your reasoning and we will seek the editor's input on an exemption.

4) Please amend your detailed Financial Disclosure statement. This is published with the article. It must therefore be completed in full sentences and contain the exact wording you wish to be published.

i) State the initials, alongside each funding source, of each author to receive each grant. For example: "This work was supported by the National Institutes of Health (####### to AM; ###### to CJ) and the National Science Foundation (###### to AM)."

ii) State what role the funders took in the study. If the funders had no role in your study, please state: "The funders had no role in study design, data collection and analysis, decision to publish, or preparation of the manuscript."

iii) If any authors received a salary from any of your funders, please state which authors and which funders..

If you did not receive any funding for this study, please simply state: u201cThe authors received no specific funding for this work.u201d

5)Please ensure that the funders and grant numbers match between the Financial Disclosure field and the Funding Information tab in your submission form. Note that the funders must be provided in the same order in both places as well. Currently, "This work was also supported by Seoul National" is missing from the Funding Information

6) We have amended your Competing Interest statement to comply with journal style. We kindly ask that you double check the statement and let us know if anything is incorrect.

**Reviewers' comments:**

Reviewer's Responses to Questions

**Comments to the Authors:**

**Please note here if the review is uploaded as an attachment.**

Reviewer #1: I recommend this article for publication with minor comments. Please find the comments attached.

Reviewer #2: The manuscript presents an insightful study that explores the connection between scale-free networks and coordinative structures, highlighting their characteristic properties. The authors propose an interesting simulation framework that demonstrates these properties and validate their predictions against published studies on motor learning.

1.In the Results section, several methodological choices are not sufficiently explained. For example, it is unclear why degree heterogeneity is selected as a key measurement. Why does a higher degree heterogeneity in the BA model indicate a better or more meaningful result? Similarly, the interpretation of the eigenvector centrality Gini coefficient is not well articulated. The authors should clarify what these metrics represent in the context of coordinative structures and how they support the main claims.

2.Since the goal is to establish a connection between scale-free networks and the characteristic properties of coordinative structures, these properties should be explicitly defined before presenting the experimental results. It would be helpful to include a summary table that outlines: (i) the specific properties of interest, (ii) the corresponding network measurements used to capture each property, (iii) the rationale for selecting these measurements, and (iv) what is the statistics calculated in the experiment. This may improve the logical flow of the paper.

3. The topic is interesting, and the simulation model appears reasonable. However, the manuscript needs to improve its readability. The current description in the Results section makes it difficult to grasp the main idea.

**********

**Have the authors made all data and (if applicable) computational code underlying the findings in their manuscript fully available?**

The PLOS Data policy requires authors to make all data and code underlying the findings described in their manuscript fully available without restriction, with rare exception (please refer to the Data Availability Statement in the manuscript PDF file). The data and code should be provided as part of the manuscript or its supporting information, or deposited to a public repository. For example, in addition to summary statistics, the data points behind means, medians and variance measures should be available. If there are restrictions on publicly sharing data or code —e.g. participant privacy or use of data from a third party—those must be specified.

Reviewer #1: Yes

Reviewer #2: Yes

**********

PLOS authors have the option to publish the peer review history of their article (what does this mean?). If published, this will include your full peer review and any attached files.

If you choose “no”, your identity will remain anonymous but your review may still be made public.

**Do you want your identity to be public for this peer review?**  For information about this choice, including consent withdrawal, please see our Privacy Policy.

Reviewer #1: No

Reviewer #2: No

[NOTE: If reviewer comments were submitted as an attachment file, they will be attached to this email and accessible via the submission site. Please log into your account, locate the manuscript record, and check for the action link "View Attachments". If this link does not appear, there are no attachment files.]

**Figure resubmission:**

While revising your submission, we strongly recommend that you use PLOS’s NAAS tool (https://ngplosjournals.pagemajik.ai/artanalysis) to test your figure files. NAAS can convert your figure files to the TIFF file type and meet basic requirements (such as print size, resolution), or provide you with a report on issues that do not meet our requirements and that NAAS cannot fix.

After uploading your figures to PLOS’s NAAS tool - https://ngplosjournals.pagemajik.ai/artanalysis, NAAS will process the files provided and display the results in the "Uploaded Files" section of the page as the processing is complete. If the uploaded figures meet our requirements (or NAAS is able to fix the files to meet our requirements), the figure will be marked as "fixed" above. If NAAS is unable to fix the files, a red "failed" label will appear above. When NAAS has confirmed that the figure files meet our requirements, please download the file via the download option, and include these NAAS processed figure files when submitting your revised manuscript.

**Reproducibility:**

To enhance the reproducibility of your results, we recommend that authors of applicable studies deposit laboratory protocols in protocols.io, where a protocol can be assigned its own identifier (DOI) such that it can be cited independently in the future. Additionally, PLOS ONE offers an option to publish peer-reviewed clinical study protocols. Read more information on sharing protocols at https://plos.org/protocols?utm_medium=editorial-email&utm_source=authorletters&utm_campaign=protocols

Attachment Submitted filename: main.pdf

# Author comment: Author response to Decision Letter 1

**DOI:** 10.1371/journal.pcbi.1014523.r002

16 Jun 2026

Attachment Submitted filename: Response to Reviewers.docx

# Peer review: Decision Letter 1

**DOI:** 10.1371/journal.pcbi.1014523.r003

**Authors:** Marc R Birtwistle, Pramod Shinde

1 Jul 2026

Dear Dr. Park,

We are pleased to inform you that your manuscript 'Coordinative structures as scale-free networks: cascade and percolation dynamics in motor learning with empirical validation' has been provisionally accepted for publication in PLOS Computational Biology.

Before your manuscript can be formally accepted you will need to complete some formatting changes, which you will receive in a follow-up email. A member of our team will be in touch with a set of requests.

Please note that your manuscript will not be scheduled for publication until you have made the required changes, so a swift response is appreciated.

IMPORTANT: The editorial review process is now complete. PLOS will only permit corrections to spelling, formatting or significant scientific errors from this point onwards. Requests for major changes, or any which affect the scientific understanding of your work, will cause delays to the publication date of your manuscript.

Should you, your institution's press office or the journal office choose to press release your paper, you will automatically be opted out of early publication. We ask that you notify us now if you or your institution is planning to press release the article. All press must be co-ordinated with PLOS.

Thank you again for supporting Open Access publishing; we are looking forward to publishing your work in PLOS Computational Biology.

Best regards,

Pramod Shinde

Guest Editor

PLOS Computational Biology

Marc Birtwistle

Section Editor

PLOS Computational Biology

***********************************************************

- Section 2.7 states that pairwise topology comparisons used two-sided t-tests, Cohen's d for effect size, and Bonferroni correction across topology pairs. I could not find any p-value, t-statistic, or effect size for any topology comparison, anywhere in the manuscript or across the Supporting Information tables (S1–S8). Please report these statistics, either in the main text where each comparison is made or in a dedicated supporting information table, with a pointer from the text.

- S1 Fig and S2 Fig are properly captioned within S1 Appendix and S4 Appendix in the Supporting Information, but the manuscript's own Supporting Information list only names “S1 Appendix” through “S6 Appendix.” Please add S1 Fig and S2 Fig as their own line items in that list, consistent with the PLOS convention of listing each Fig separately from each appendix. This is not required before acceptance.

Reviewer's Responses to Questions

**Comments to the Authors:**

**Please note here if the review is uploaded as an attachment.**

Reviewer #1: The authors have satisfactorily addressed my concerns, and I am happy to recommend this article for publication.

Reviewer #2: The latest version has improved readability a lot. I have no further questions.

**********

**Have the authors made all data and (if applicable) computational code underlying the findings in their manuscript fully available?**

The PLOS Data policy requires authors to make all data and code underlying the findings described in their manuscript fully available without restriction, with rare exception (please refer to the Data Availability Statement in the manuscript PDF file). The data and code should be provided as part of the manuscript or its supporting information, or deposited to a public repository. For example, in addition to summary statistics, the data points behind means, medians and variance measures should be available. If there are restrictions on publicly sharing data or code —e.g. participant privacy or use of data from a third party—those must be specified.

Reviewer #1: Yes

Reviewer #2: None

**********

PLOS authors have the option to publish the peer review history of their article (what does this mean?). If published, this will include your full peer review and any attached files.

If you choose “no”, your identity will remain anonymous but your review may still be made public.

**Do you want your identity to be public for this peer review?**  For information about this choice, including consent withdrawal, please see our Privacy Policy.

Reviewer #1: **Yes:** Saptarshi Ghosh

Reviewer #2: No

# Editor report: Acceptance letter

**DOI:** 10.1371/journal.pcbi.1014523.r004

**Authors:** Marc R Birtwistle, Pramod Shinde

PCOMPBIOL-D-26-00505R1

Coordinative structures as scale-free networks: cascade and percolation dynamics in motor learning with empirical validation

Dear Dr Park,

I am pleased to inform you that your manuscript has been formally accepted for publication in PLOS Computational Biology. Your manuscript is now with our production department and you will be notified of the publication date in due course.

The corresponding author will soon be receiving a typeset proof for review, to ensure errors have not been introduced during production. Please review the PDF proof of your manuscript carefully, as this is the last chance to correct any errors. Please note that major changes, or those which affect the scientific understanding of the work, will likely cause delays to the publication date of your manuscript.

Soon after your final files are uploaded, unless you have opted out, the early version of your manuscript will be published online. The date of the early version will be your article's publication date. The final article will be published to the same URL, and all versions of the paper will be accessible to readers.

For Research, Software, and Methods articles, you will receive an invoice from PLOS for your publication fee after your manuscript has reached the completed accept phase. If you receive an email requesting payment before acceptance or for any other service, this may be a phishing scheme. Learn how to identify phishing emails and protect your accounts at https://explore.plos.org/phishing.

Thank you again for supporting PLOS Computational Biology and open-access publishing. We are looking forward to publishing your work!

With kind regards,

Kannan R K Kuppusamy, B.TECH BIOTECHNOLOGY

PLOS Computational Biology | Carlyle House, Carlyle Road, Cambridge CB4 3DN | United Kingdom ploscompbiol@plos.org | Phone +44 (0) 1223-442824 | ploscompbiol.org | @PLOSCompBiol
